# Weighted Blind Source Separation Can Decompose the Frequency Mismatch Response by Deviant Concatenation: An MEG Study

**DOI:** 10.3389/fneur.2022.762497

**Published:** 2022-02-25

**Authors:** Teppei Matsubara, Steven Stufflebeam, Sheraz Khan, Jyrki Ahveninen, Matti Hämäläinen, Yoshinobu Goto, Toshihiko Maekawa, Shozo Tobimatsu, Kuniharu Kishida

**Affiliations:** ^1^Athinoula A. Martinos Center for Biomedical Imaging, Massachusetts General Hospital, Charlestown, MA, United States; ^2^Harvard Medical School, Boston, MA, United States; ^3^Japan Society for the Promotion of Science, Tokyo, Japan; ^4^International University of Health and Welfare, Fukuoka, Japan; ^5^Department of Physiology, School of Medicine, International University of Health and Welfare, Narita, Japan; ^6^Department of Psychiatry, Amekudai Hospital, Naha, Japan; ^7^Department of Orthoptics, Faculty of Medicine, Fukuoka International University of Health and Welfare, Fukuoka, Japan; ^8^Gifu University, Gifu, Japan; ^9^Hermitage of Magnetoencephalography, Osaka, Japan

**Keywords:** mismatch response (MMR), blind source separation (BSS), magnetoencephalography (MEG), time-delayed correlation, independent component analysis (ICA), *T/k* (fractional) type of decorrelation method, weighted blind source separation, deviant concatenation

## Abstract

The mismatch response (MMR) is thought to be a neurophysiological measure of novel auditory detection that could serve as a translational biomarker of various neurological diseases. When recorded with electroencephalography (EEG) or magnetoencephalography (MEG), the MMR is traditionally extracted by subtracting the event-related potential/field (ERP/ERF) elicited in response to “deviant” sounds that occur randomly within a train of repetitive “standard” sounds. However, there are several problems with such a subtraction, which include increased noise and the neural adaptation problem. On the basis of the original theory underlying MMR (i.e., the memory-comparison process), the MMR should be present only in deviant epochs. Therefore, we proposed a novel method called weighted-*BSS*_*T*/k_, which uses only the deviant response to derive the MMR. Deviant concatenation and weight assignment are the primary procedures of weighted-*BSS*_*T*/k_, which maximize the benefits of time-delayed correlation. We hypothesized that this novel weighted-*BSS*_*T*/k_ method highlights responses related to the detection of the deviant stimulus and is more sensitive than independent component analysis (ICA). To test this hypothesis and the validity and efficacy of the weighted-*BSS*_*T*/k_ in comparison with ICA (infomax), we evaluated the methods in 12 healthy adults. Auditory stimuli were presented at a constant rate of 2 Hz. Frequency MMRs at a sensor level were obtained from the bilateral temporal lobes with the subtraction approach at 96–276 ms (the MMR time range), defined based on spatio-temporal cluster permutation analysis. In the application of the weighted-*BSS*_*T*/k_, the deviant responses were given a constant weight using a rectangular window on the MMR time range. The ERF elicited by the weighted deviant responses demonstrated one or a few dominant components representing the MMR that fitted well with that of the sensor space analysis using the conventional subtraction approach. In contrast, infomax or weighted-infomax revealed many minor or pseudo components as constituents of the MMR. Our single-trial, contrast-free approach may assist in using the MMR in basic and clinical research, and it opens a new and potentially useful way to analyze event-related MEG/EEG data.

## Introduction

The mismatch negativity component in electroencephalography (EEG), and its magnetoencephalographic (MEG) counterpart the mismatch field (or mismatch response, MMR), are event-related responses (EPRs/ERFs) widely used to measure auditory processing in cognitive neuroscience (1–6). The MMR is recorded using an oddball paradigm, where the repeated presentation of a stimulus (standard) is occasionally replaced by a different stimulus (deviant). The MMR is then computed as the difference between the deviant and standard responses. This difference representing the MMR is typically found around 100–250 ms after the onset of the deviant stimulus (7). Previous studies have revealed a cortical network consisting mainly of the bilateral temporal regions, but also the frontal and parietal regions, which is involved in the generation of the MMR (8–10). The prevailing view is that the MMR reflects the detection of change in the auditory system that can be measured without attention, although alternative interpretations exist (11–14). The MMR has therefore been widely used to assess auditory processing in children and clinical groups (10, 15, 16).

Originally, it was suggested that the occurrence of the MMR relates to the presence of a short-term memory trace where the memory-comparison process detects a discrepancy between the neural representation of the regularity inherent in the recent stimulation and the representation of the current deviant stimulus (17). On the basis of this hypothesis, obtaining a difference waveform by subtracting the standard response from the deviant response is the only way to identify the MMR. However, there are several problems associated with the subtraction approach. First, the subtraction reduces the signal-to-noise ratio (SNR) because the noise present in the standard responses is added to the noise in the deviant responses. Second, the neural adaptation process, especially with frequency MMR, can affect the difference waveform. The auditory system has a tonotopic organization from the cochlea through to the cortex (18). Stimulus repetition leads to repeated initiation of patterns of neural activity (e.g., the M100) that habituates as a function of the repetition rate (19, 20). In the classic oddball protocol, the neural response to standard stimuli is attenuated by these repetition suppression effects. This suppression is greater for the standard stimuli than for the less frequent deviant stimuli. The adapted and non-adapted neural activity presents not only different amplitudes, but also different temporal dynamics. Thus, the subtraction approach does not simply reflect the MMR (i.e., a memory-based comparison) but also the differential adaptation of neurons (13). Therefore, the study of the temporal dynamics of the MMR might convey critical information regarding the nature of the underlying neural generators. Hence, to effectively reveal the MMR, another approach considering the temporal information, instead of the subtraction approach, is desirable.

Each EEG electrode or MEG sensor records a linear combination of signals from several sources (21). Multi-channel EEG/MEG, which typically involves hundreds of sensors, provides detailed spatio-temporal distribution patterns, which obviously complicate the interpretation of signals and topographies. Independent component analysis (ICA), which is a blind source separation (BSS) method, is a stochastic method that can be used to decompose such complex data into a set of spatio-temporal components, each of which comprises a fixed spatial distribution and an associated signal (22, 23). Each component signal is a weighted sum of the sensor or electrode signals, which in turn are weighted sums of the dynamics of the neural sources (24). ICA/BSS can provide signal sources without any a priori information about their occurrence in biological signals. In general, the single-trial approach of ICA/BSS can utilize temporal information, because the contraction of information occurs during the averaging process of the ERP/ERF. A single trial may contain all kinds of non-brain artifacts and spontaneous EEG/MEG processes, whereas decomposing an average of all trials not only minimizes the contributions of those neural and artifactual processes that are not reliably time- and phase-locked to experimental events but also removes event-related brain dynamics among trials (25). As artifacts often exhibit stereotypical patterns that differ from those of brain activity, ICA/BSS can mostly be used to separate artifactual patterns (26–28). In fact, ICA/BSS has been used to extract event-related activities in only a handful of previous studies (29–33). Owing to the components being computed based purely based on their statistical independence, physiological perspectives are not taken into account (28, 34). Considering that regional brain activities substantially correlate with each other, an approach requiring strong independence may not be the most fruitful (35–38).

An approach for refining ICA/BSS using time-delayed correlation, or the decorrelation method (DC) has also been considered (39). Time-delayed correlation takes account of the characteristic time structure of the signals of interest, including the periodicity and/or morphology. Thus, time-delayed correlation measures the correlation between two signals, then maximizes the correlation between components. For example, several studies applied DC to second-order blind identification (SOBI) to separate periodic signals, such as cardiac and oscillatory brain activity, because periodic signals are well-correlated with delayed signals and non-delayed original signals (40, 41). As a result, well-correlated signals were extracted in one (or a few) components. However, in SOBI, most approaches examine the time structure of the target signals subjectively. When parameters are highly specified, featured components are more independent, and therefore target signals collapse because of strong independence and the SOBI method becomes equivalent to ICA (39–41).

In an attempt to develop solutions to address the limitations of ICA and SOBI, we proposed a novel method of BSS called the *T/k* (fractional) type of DC (*BSS*_*T*/k_) (35–38). This method shares the fundamental concept underlying DC such as SOBI but is more focused on the periodicity of the target signal. The *BSS*_*T*/k_ method is based on extracting time points (i.e., time-delayed parameters) determined by the parameters *T* and *k*, which represent periodicity concerning a fundamental and harmonics (Supplementary Data (1) and Supplementary Figure 1). *BSS*_*T*/k_ allows weak independence among the components. Setting time-delayed parameters in this way results in highlighting the characteristics of target ERFs that are periodically presented. Previously, we demonstrated that somatosensory-evoked fields in response to periodic electrical stimuli can be decomposed into a few components using the *BSS*_*T*/k_ algorithm in 64 channel magnetometers of CTF (35–38). Using a generalization of *BSS*_*T*/k_, non-periodic interictal epileptiform discharges that were assumed to originate in a single epileptogenic zone were decomposed into one dominant component (42).

For the MMR paradigm, where deviant stimuli are presented in random order, we proposed to use a modification of *BSS*_*T*/k_, which we termed weighted-*BSS*_*T*/k_ (43). In weighted-*BSS*_*T*/k_, we only used deviant responses that were concatenated into a periodical arrangement. Then, deviant responses were assigned a constant weight (rectangular window) on the specific time interval that represents MMR (i.e., the MMR time range). This is known as a window function in the time domain. The MMR time range was defined in a data-driven manner using sensor space subtraction (i.e., the reference standard). Through these procedures, the correlation between MMR and the responses outside of the MMR time range (e.g., the M100) can be minimized; thus, weighted-*BSS*_*T*/k_, which underlies time-delayed correlation, can effectively extract the MMR. We hypothesized that weighted-*BSS*_*T*/k_ would extract one or a few dominant components that can discriminate the MMR from background brain noise and other artifacts or other irrelevant ERFs. As the first application in the cognitive neuroscience of weighted-*BSS*_*T*/k_ using only deviant epochs, we aimed to extract components that resemble the reference standard because subtraction is currently the gold standard for identifying MMR. We applied both *BSS*_*T*/k_ and infomax (ICA) separately to the same weighted multi-channel MEG data (weighted-*BSS*_*T*/*k*_ and weighted-infomax, respectively), and used the subtraction approach (subtraction-*BSS*_*T*/*k*_ and subtraction-infomax) as a more general approach to investigate how the single-trial approach works, and then, statistically compared the similarity of each component to the reference standard to test a further hypothesis that *BSS*_*T*/k_ is more sensitive than infomax.

It was not our aim to use the subtraction-*BSS*_*T*/k_/weighted-*BSS*_*T*/k_ to separate independent MMR sources. Typically, statistically independent components separated by preprocessing with ICA are expected to be associated with one or two dipolar sources (9, 23, 44, 45). We instead made a more general assumption that a component extracted by subtraction-*BSS*_*T*/k_/weighted-*BSS*_*T*/k_ will relate to multiple sources or a network of activity generating the MMR. In this sense, few decomposed components are better than many, as long as they represent the reference standard. Thus, the extraction of MMR in a few components would simplify the interpretation of MMR in regard to clinical and research applications.

## Materials and Methods

### Participants

The participants in the experiment were 12 healthy adults (aged 25.4–41.9 years, mean 33.7 years; six women). None of the participants reported a history of head injury, neurological disease, hearing problems, severe medical illness, or drug abuse. The experiment was approved by the Ethics Committee of Kyushu University.

### Stimuli and Procedures

The paradigm consisted of auditory stimulus sequences composed of standard stimuli with a probability of 80% and deviant stimuli with a probability of 20%, which were delivered in random order until at least 150 deviant stimuli were presented. Tone bursts of 500 Hz for standard stimuli and 550 Hz for deviant stimuli (10-ms rise and 20-ms fall) with a 100-ms duration were delivered monaurally through plastic tubes (length, 6 m; inner diameter, 8 mm). The hearing threshold was determined for each ear of each subject, and stimuli generated by a tone-burst-generator (Kyushu-Keisokuki, Fukuoka, Japan) were delivered at intensities of 50 dB above the threshold (46). The stimulus onset asynchrony (SOA) was 500 ms, and the presentation rate of the stimuli represented by *f*_*p*_ was 2 Hz. Stimuli were delivered to each ear in separate runs, with masking noises delivered to the contralateral ear to avoid cross-hearing (47). Inversed stimuli (550 Hz for standard and 500 Hz for deviant) were presented monaurally in separate runs. These stimuli were counterbalanced. In the current study, only data from right-ear stimulation and using 500-Hz standard/550-Hz deviant stimuli were analyzed. Subjects were instructed to ignore the auditory stimuli while they lay on the bed and watched a silent movie (16).

### Data Acquisition

MEG was acquired using a 306-channel (204 planar gradiometers and 102 magnetometers) whole-head system (Elekta-Neuromag, Helsinki, Finland) in a magnetically shielded room. The sampling rate was 1,000 Hz, with a band-pass filter of 0.03–330 Hz. EEG was simultaneously recorded using 19 scalp electrodes according to the international 10–20 system, although the sparse EEG data were not analyzed in the current study.

### Data Analysis

#### Preliminary Process

**Figure 1:**
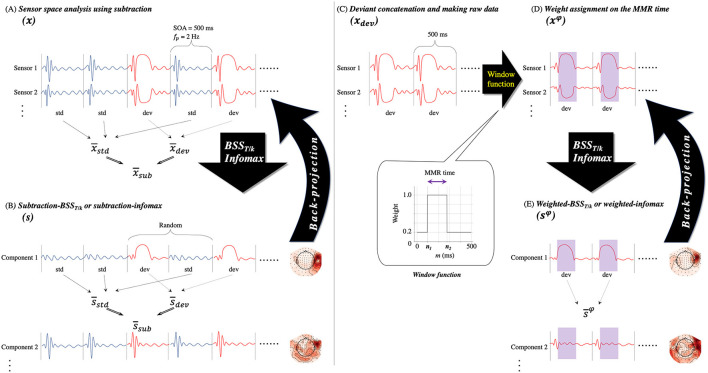
Analysis steps. **(A)** The conventional subtraction approach for sensor space analysis. Sensor data (*x*) consist of standard (std) and deviant (dev) epochs. The MMR difference sensor waveform (${\bar{x}}_{sub}$) is calculated by subtracting the event-related field (ERF) to the standard (${\bar{x}}_{std}$) stimulus from the ERF to the deviant (${\bar{x}}_{dev}$) stimulus. *BSS*_*T*/*k*_, fractional type of decorrelation method; MMR, mismatch response; SOA, stimulus onset asynchrony; *f*_*p*_, presentation rate of stimuli. **(B)** The subtraction approach with two decomposition methods (*BSS*_*T*/*k*_ or infomax). Data from 204 sensors (*x*) are decomposed into 204 components (s). The subtraction approach is followed for each component; subtraction of the deviant ERF (${\bar{s}}_{dev}$) from the standard ERF (${\bar{s}}_{std}$) makes the MMR difference source waveform (${\bar{s}}_{sub}$). Note that deviant epochs occur randomly, not periodically. **(C)** Deviant epochs of sensor data are concatenated and new raw data are made (*x*_*dev*_). This process makes periodical arrangements of deviant epochs. **(D)** Sensor data (*x*^φ^) are assigned with a weight on the MMR time (from *n*_1_ to *n*_2_, highlighted in purple shadows) on *x*_*dev*_ using a window function. The window function is shown in the inset figure. **(E)** Data with 204 sensors assigned with a weight (*x*^φ^) are decomposed into 204 components (*s*^φ^) with two decomposition methods. In each component, the ERF of the deviant epochs assigned with a weight is obtained (${\bar{s}}^{\varphi }$). The inverted black arrows between A and B and between D and E represent the back-projection process in a group of several components.

**Figure 2:**
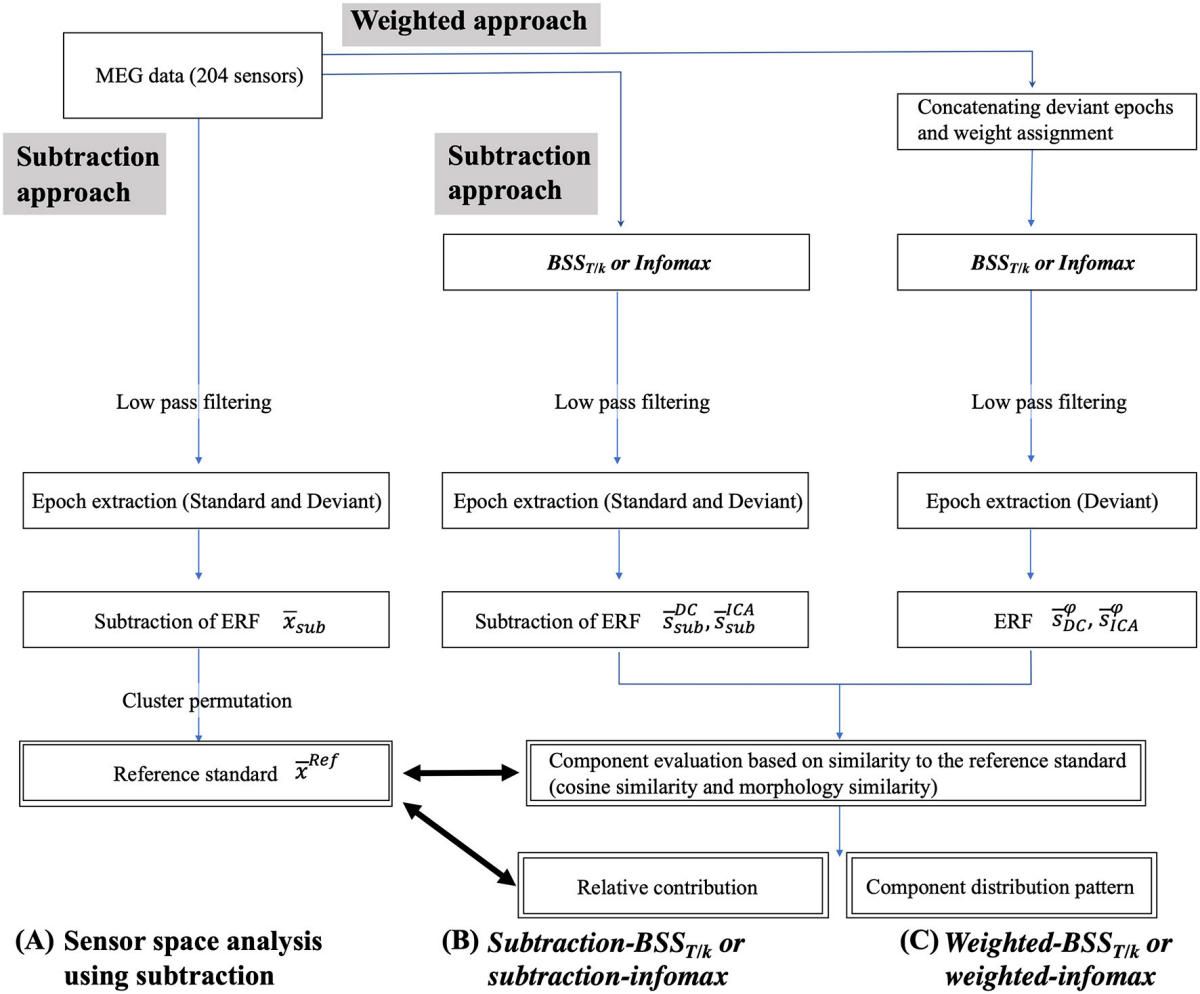
Block diagram of the procedure for the subtraction **(A,B)** and weighted approaches **(C)** for each decomposition method (*BSS*_*T*/*k*_ or infomax). The main analysis parts are shown in double squares. *BSS*_*T*/*k*_, fractional type of decorrelation method; DC, decorrelation method; ERF, event-related field; ICA, independent component analysis.

The temporal signal space separation method (TSSS) using MaxFilter 2.2.13 (Elekta-Neuromag, Helsinki, Finland) was applied to the sensor level data with the default setting of an inside expansion order of 8, an outside expansion order of 3, automatic optimization of both inside and outside bases, a subspace correlation limit of 0.980, and a raw data buffer length of 10 s (48, 49). Notch filters were applied to suppress power line frequency and its harmonics (60, 100, 120, 180, 200, 240, and 300 Hz). Data from the 204 planar gradiometers were used for all subsequent analyses. Hereafter, all analysis steps are shown in Figure 1 and summarized in Figure 2.

#### The Subtraction Approach

The conventional subtraction approach for sensor space analysis was used as a reference (Figures 1A, 2A). Before averaging across epochs, the data were low pass filtered at 30 Hz and, epochs exceeding 4,000 fT/cm on any planar gradiometer channel were excluded from the average. Based on our experience, some ocular artifacts leak into the good epochs. Therefore we took extra care and visually inspected the data to remove eye movements. However, the impact of this procedure was minimal because the number of epochs removed for each subject was 0–1. Each epoch contained a 600-ms time window ranging from 100 ms pre-stimulus to 500 ms post-stimulus onset, with the stimuli being periodically presented (*SOA* = 500 ms or *f*_*p*_ = 2 Hz). The MMR difference sensor waveform (i.e., ${\bar{x}}_{sub}$) was calculated by subtracting the averaged deviant ERFs from the averaged standard ERFs for each subject (Figures 1A, 2A);


(1) $$\begin{array}{l} {\bar{x}}_{sub}\left(n\right)\;:=\;{\bar{x}}_{dev}\left(n\right)-{\bar{x}}_{std}\left(n\right), \end{array}$$

where *x*(*n*) represents the MEG sensor data at the discrete time, *n*. $\bar{x}\left(n\right)$ reflects the averaged sensor waveform of *x*(*n*) across epochs. ${\bar{x}}_{std}\left(n\right)$ and ${\bar{x}}_{dev}\left(n\right)\;$are averaged standard and deviant responses, respectively.

#### Decomposition Process

The decomposition methods of *BSS*_*T*/*k*_ and infomax were applied separately to each subject's sensor dataset, which contained 204 sensors. The sensor data were originally decomposed into a set of spatio-temporal components;


(2) $$\begin{array}{l} x\left(n\right)=As\left(n\right), \end{array}$$

where *A* is a mixing matrix, and *s* is a signal source. *BSS*_*T*/*k*_ was applied;


(3) $$\begin{array}{l} x\left(n\right)=A_{DC}s_{DC}\left(n\right), \end{array}$$

where *A*_*DC*_ is a mixing matrix of *BSS*_*T*/*k*_, and *s*_*DC*_ is a signal source of *BSS*_*T*/*k*_. Hereafter, we refer to BSSq (q = 1, 2, 3, …, 204) as a specific component obtained after the application of *BSS*_*T*/*k*_. We briefly describe the *BSS*_*T*/*k*_ method here; full details are provided in previous studies (36, 38). As a preliminary step, we conducted a sphering procedure to orthogonalize and normalize the time-series data for input sensors. We then conducted an iterative Givens rotation to minimize the absolute sum of off-diagonal elements of the normalized correlation matrices at the parameters. Specifically, the Jacobi-like algorithm proposed by Cardoso and Souloumiac (50, 51) was used in the *BSS*_*T*/*k*_ method to approximately solve the simultaneous diagonalization problem at specific times. Regarding the period *T* = *1/f*_*p*_ with sampling frequency *f*_*s*_, the time-delayed parameters τ can be defined by:


(4) $$\begin{array}{l} BSS_{T/k}:\tau_{m}=\left[f_{s}/f_{p}\right]/m,\;m=1,2,\ldots ,k. \end{array}$$

where *[…]* rounds the value to the nearest integer. Here, *T* = 0.5 s and *f*_*p*_ = 2 Hz, with the repetitive stimuli constantly presented at a rate of 2 Hz (subsection Stimuli and Procedures). We determined *k* = 8 in a data-driven manner (36, 38) [Supplementary Data (1) and Supplementary Figure 1]. These parameters gave τ (ms) as 500, 250, 166, 125, 100, 83, 71, and 62 according to Eq. (4).

For ICA, we used the infomax algorithm (25, 49), which was implemented in MNE-python (52) using the default setting;


(5) $$\begin{array}{l} x\left(n\right)=A_{ICA}s_{ICA}\left(n\right), \end{array}$$

where *A*_*ICA*_ is the mixing matrix of infomax, and *s*_*ICA*_ is the signal source of infomax. Hereafter, we refer to ICAq (q = 1, 2, 3, …, 204) as a specific component obtained after application of infomax. The number of principal components from the pre-whitening step that was passed to the ICA algorithm was 204, which corresponded with the number of sensor inputs. Accordingly, we obtained 204 components with associated time courses and spatial distributions.

#### Two Different Approaches (Subtraction and Weighted)

After applying the decomposition methods (*BSS*_*T*/*k*_ and infomax) to the sensor space data, we obtained the MMR difference source waveform (i.e., ${\bar{s}}_{sub}$; Figures 1B, 2B) in the same way as in the subtraction approach for sensor space analysis [subsection The Subtraction Approach; Eq. (1); Figures 1A, 2A], which corresponds to the two decomposition methods (i.e., subtraction-*BSS*_*T*/*k*_ and subtraction-infomax; Figures 1B, 2B);


(6) $$\begin{array}{l} {\bar{s}}_{sub}^{DC}\left(n\right):={\bar{s}}_{dev}^{DC}\left(n\right)-\;{\bar{s}}_{std}^{DC}\left(n\right), \end{array}$$

(7) $$\begin{array}{l} {\bar{s}}_{sub}^{ICA}\left(n\right):={\bar{s}}_{dev}^{ICA}\left(n\right)-\;{\bar{s}}_{std}^{ICA}\left(n\right), \end{array}$$

where ${\bar{s}}_{std}\left(n\right)$ and ${\bar{s}}_{dev}\left(n\right)$ are the averaged source waveforms across epochs (i.e., ERFs) elicited by the standard and deviant stimulus, respectively, obtained from each decomposition method.

The novel method, the weighted-*BSS*_*T*/*k*_, is expected to be a more sensitive approach of extracting the MMR. The basics of the method lie in the periodical arrangements and assignments of weights on the MMR time range. Although our *BSS*_*T*/*k*_ method is expected to highlight periodic signals, the deviant epochs occur randomly, not periodically. To obtain periodical arrangements, we concatenated the deviant epochs to form new raw data (*x*_*dev*_(*n*); Figure 1C). To highlight the MMR that was included in the deviant epochs, we then weighted the MMR time range (around 100–250 ms, from *n*_1_ to *n*_2_) defined by the spatio-temporal cluster permutation (subsection Spatio-Temporal Cluster Permutation to Define the MMR Time Ranges and Sensors or the Reference Standard), with the weight described by the window function of the rectangular window (inset between Figures 1C,D);


(8) $$\begin{array}{l} x^{\varphi }\left(n\right):=\varphi *x_{dev}\left(n\right), \end{array}$$

where φ describes a window function and the ^*^ reflects its repeat operation. The segmentation of data (epoch number, mean 174.3 ± 19.6 [standard deviation]) was multiplied by the window function values. Equation (8) indicates,


(9) $$\begin{array}{l} \{\begin{array}{c} \;x^{\varphi }\left(n\right)=1\cdot x_{dev}\left(n\right),\;n_{1}\;\leq \;m\;\leq \;n_{2},\\ x^{\varphi }\left(n\right)=0.2\cdot x_{dev}\left(n\right),\;m<\;n_{1},\;n_{2}<m, \end{array} \end{array}$$

where *n* = (*Index of deviant epoch* − 1)·*SOA* + *m*. Here, *m* is the given time point within every deviant epoch. Equation (8) indicates that this window function, Eq. (9), was applied repeatedly (Figure 1D, purple shadow) to the concatenated sensor data (*x*_*dev*_(*n*)). We then applied the *BSS*_*T*/*k*_ and infomax methods separately to the weighted data (weighted-*BSS*_*T*/*k*_ and weighted-infomax; Figure 1E);


(10) $$\begin{array}{l} x^{\varphi }\left(n\right)=A_{DC}^{\varphi }s_{DC}^{\varphi }\left(n\right), \end{array}$$

(11) $$\begin{array}{l} x^{\varphi }\left(n\right)=A_{ICA}^{\varphi }s_{ICA}^{\varphi }\left(n\right). \end{array}$$

Finally, after lowpass filtering (30 Hz), we obtained the ERFs (i.e., ${\bar{s}}^{\varphi }$; Figures 1E, 2C). That is, ${\bar{s}}_{DC}^{\varphi }\left(n\right)$ and ${\bar{s}}_{ICA}^{\varphi }\left(n\right)$, elicited by the weighted deviant stimulus, instead of subtraction.

Two assumptions underlie the successful decomposition of the weighted-*BSS*_*T*/*k*_. First, the MMR occurs in the MMR time (*n*_1_ ≤ *n* ≤ *n*_2_) only in deviant epochs. Second, exogenous/obligatory ERFs (e.g., the M100) highly correlate with themselves in the non-MMR time (*n* < *n*_1_, *n*_2_ < *n*). The offset response of the M100 often intrudes on the MMR within the MMR time, which is one of the reasons why the subtraction approach is necessary (53). To minimize the joint M100 and MMR effect, a rectangular window in the non-MMR time is used to keep the correlation of the offset and onset of the M100 and extract these as distinct components from an MMR component using weighted-*BSS*_*T*/*k*_, which underlies time-delayed correlation. However, it is expected that weighted-infomax, in contrast to weighted-*BSS*_*T*/*k*_, does not decompose the MMR effectively because infomax does not depend on time structure.

#### Spatio-Temporal Cluster Permutation to Define the MMR Time Ranges and Sensors or the Reference Standard

**Figure 3:**
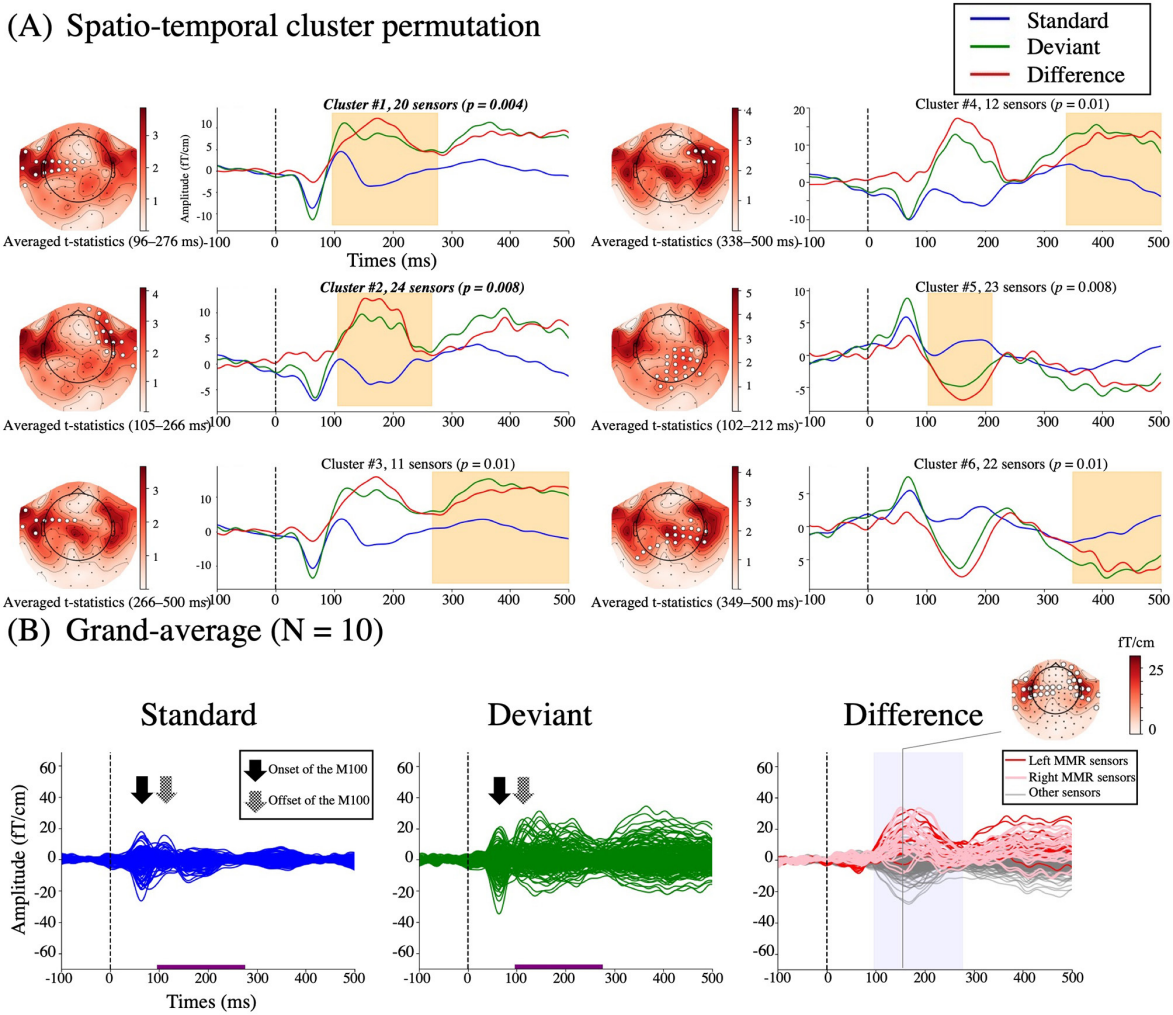
Sensor space waveform. **(A)** The results of the spatio-temporal cluster permutation analysis. Six significant spatial and temporal clusters are shown in white circles within the averaged t-statistics (absolute value) and in orange shading within the averaged waveforms, respectively. Blue lines, standard; green lines, deviant; red lines, difference. **(B)** Grand-averaged ERFs elicited by standard (${\bar{x}}_{std}$) and deviant (${\bar{x}}_{dev}$) stimuli and the MMR difference sensor waveform (${\bar{x}}_{sub}$) from 10 subjects. The MMR time is indicated by the purple line in the standard and deviant ERFs and by the blue shading in the MMR difference sensor waveform. The red and pink lines in the MMR difference sensor waveform represent the MMR sensors from the left and right clusters, respectively. The topographical map represents the peak activity in the bilateral temporal and right frontal sensors (white circles). The onset of the M100 (arrows) is outside of the MMR time range, whereas the offset of the M100 (textured arrows) is included in the MMR time range seen in standard and deviant responses.

Currently, the only way to identify MMR is via sensor-space subtraction. We therefore used sensor-space subtraction as a reference standard. A data-driven approach was used to find significant MMR time ranges and sensors in all subjects. Among the 12 subjects, two did not exhibit a prominent MMR during the initial screening of the visual inspection of sensor space subtraction (confirmed by three independent inspectors, TMat, SK, and KK.) and were thus excluded from further analysis. Individual MMR difference sensor waveforms, ${\bar{x}}_{sub}$, were tested if they were different from 0 across the 10 subjects, with the multiple comparison problem being addressed using a cluster-level permutation test across space and time (54). We used 1,024 permutations, and the cluster-defining threshold was set at *p* = 0.01. Selected samples were clustered based on both spatial and temporal adjacency (i.e., spatio-temporal cluster permutation). Our motivation to use the spatio-temporal cluster permutation method was to verify the empirical knowledge that MMR occurs around 100–250 ms in the bilateral front-temporal sensors (7, 17) in a data-driven manner in our cohort of 10 subjects. Figure 3A demonstrates the results of the spatio-temporal cluster permutation. Six clusters (less than the critical alpha level of 0.05) were found. Among these six clusters, two (#1 and #2) contained temporal and/or frontal sensors within approximately 100–250 ms; one (#1) contained 20 left temporal sensors at 96–276 ms and the other (#2) contained 24 right front-temporal sensors at 105–266 ms. Thus, we defined the MMR time range as 96–276 ms (*n*_1_ = 96, *n*_2_ = 276) and the MMR sensors as these 44 sensors. The reference standard was defined individually (Figure 2A);


(12) $$\begin{array}{l} {\bar{x}}^{Ref}:=\;F{\bar{x}}_{sub}\left(n\right),\;n_{1}\leq n\leq n_{2}, \end{array}$$

where ${\bar{x}}^{Ref}\in \;{\mathbb{R}}^{L\times \left(n_{2}-n_{1}\right)}$ and *F* ∈ ℝ^*L*×*N*^ is the matrix that select *L* = 44 rows corresponding to the MMR sensors out of ${\bar{x}}_{sub}$ containing all *N* = 204 sensors. In other words, the reference standard was the 44 MMR sensors selected from the 204 gradiometers within the MMR time.

We confirmed that the different setting of the cluster-defining threshold (*p* = 0.005) gave the similar spatio-temporal clusters (Supplementary Figure 2). This means that the clusters obtained were robust.

#### Component Evaluation: Cosine Similarity

To investigate the resemblance of each component to the reference standard individually, or goodness of fit, we measured cosine similarity (*C*) as spatial similarity and morphology similarity (*M*) as temporal similarity.

Cosine similarity refers to the similarity between two column vectors (42, 55);


(13) $$\begin{array}{l} \text{Cosine similarity }\left(C\right):\;C\left(a\left(n\right),b\right)=\left|{â\left(n\right)}^{T}\hat{b}\right|,n_{1}\leq n\leq n_{2}, \end{array}$$

where â(*n*) = *a*(*n*)/|*a*| is the normalized column vector containing the spatial distribution of the reference standard (${\bar{x}}^{Ref}$), and $\hat{b}=b/|b|\;$is a normalized column vector of *A* in Eqs. (3, 5, 10, and 11). The symbol *T* is the transpose of â(*n*). Because of its definition, 0 ≤ *C*(*a*(*n*), *b*) ≤ 1. In the following, we used the maximum of *C* (*C*_max_) across the MMR time range for the four methods (i.e., subtraction-*BSS*_*T*/*k*_, subtraction-infomax, weighted-*BSS*_*T*/*k*_, and weighted-infomax), denoted by $C_{\max}^{DC}$, $C_{\max}^{ICA}$, $C_{\max}^{DC\text{\_}\varphi }$, and $C_{\max}^{ICA\text{\_}\varphi }$. *C*_max_ represents how maximally similar each component is to the reference standard in regard to spatial information.

#### Component Evaluation: Back-Projection and Morphology Similarity

Temporal similarity should include information about the temporal correlation between each component and the reference standard as well as the amplitude difference between each component and the reference standard. Because the components derived from *BSS*_*T*/*k*_ and infomax ($M_{DC}\left(n\right),\;M_{ICA}\left(n\right){,\;M}_{DC}^{\varphi }\left(n\right),$ and $M_{ICA}^{\varphi }\left(n\right)$) are differently normalized, their ERFs cannot be directly compared according to their amplitudes. Thus, each component was projected back into the sensor space (back-projection) (56). Here, we assumed a general situation for the sake of the following subsection The Cumulative Back-Projection of Salient Components, the cumulative back-projection. When a group of q components, where Q = {q} is selected from 204 components,


(14) $$\begin{array}{l} x_{\#}^{q}\left(n\right)=\;A_{\#}^{q}s_{\#}^{q}\left(n\right) \end{array}$$

provides back-projected data in the sensor space (inverted black arrow between Figures 1A,B), where $A_{\#}^{q}\;\in {\mathbb{R}}^{204\times q}$ and $s_{\#}^{q}\left(n\right)$ represents source vectors corresponding to Q. Here, the suffix symbol # indicates DC or ICA. The same formula was applied to the weighted data (inverted black arrow between Figures 1D,E). The ERF was then computed using the subtraction or weighted approach. For the subtraction approach, we applied


(15) $$\begin{array}{l} {\bar{x}}_{\#\text{\_}sub}\left(n,q\right):={\bar{x}}_{\#\text{\_}dev}\left(n,\;q\right)-{\bar{x}}_{\#\text{\_}std}\left(n,\;q\right), \end{array}$$

where ${\bar{x}}_{\#\text{\_}std}\left(n,q\right)$ and ${\bar{x}}_{\#\text{\_}dev}\left(n,q\right)\;$are ERFs in the sensor space elicited by standard and deviant stimuli, respectively, obtained from each decomposition method (DC or ICA). For the weighted approach$,\;{\bar{x}}_{\#}^{\varphi }\left(n,q\right)$ is the ERF obtained from each decomposition method (DC or ICA). Then, corresponding to Eq. (12), we applied


(16) $$\begin{array}{l} {\bar{x}}_{\#\text{\_}sub}\left(q\right):=F{\bar{x}}_{\#\text{\_}sub}\left(n,q\right),\;n_{1}\leq n\leq n_{2}, \end{array}$$

(17) $$\begin{array}{l} {\bar{x}}_{\#}^{\varphi }\left(q\right):=F{\bar{x}}_{\#}^{\varphi }\left(n,q\right),\;n_{1}\leq n\leq n_{2}, \end{array}$$

where ${\bar{x}}_{\#\text{\_}sub}\left(q\right)\;and\;{\bar{x}}_{\#}^{\varphi }\in {\mathbb{R}}^{L\times \left(n_{2}-n_{1}\right)}$.

We investigated the correlation between one sensor and the reference standard;


(18) $$\begin{array}{l} r_{l}=\frac{\left(X_{l},Y_{l}\right)}{\left\|X_{l}\|\|Y_{l}\right\|},\text{l}=1,2,3,\ldots ,44. \end{array}$$

where *(X, Y)* is the inner product. Here, *X* is one row vector (*l*) of the reference standard (${\bar{x}}^{Ref}$), which corresponds to one sensor, and *Y* is one row vector (*l*) of the same sensor of Z, where *Z*(*q*) is defined as Eq. (16) or Eq. (17). Notably, ${\bar{x}}_{\#\text{\_}sub}\left(n,\;q\right)\;and\;{\bar{x}}_{\#}^{\varphi }\left(n,q\right)\;\in {\mathbb{R}}^{L\times SOA}$ and $Z\left(q\right)\;\in {\mathbb{R}}^{L\times \left(n_{2}-n_{1}\right)}$. Equation (18) is the same formula as that for the Pearson coefficient. Then,


(19) $$\begin{array}{l} \text{Morphology similarity }\left(M\right):\;r_{l}\|Y_{l}\|=\frac{\left(X_{l},Y_{l}\right)}{\left\|X_{l}\right\|}\text{l}=1,2,3,\ldots ,44. \end{array}$$

was applied to calculate morphology similarity (*M*), where *M* is the comparison of the similarity of the waveforms between the reference standard and back-projected waveforms regarding the temporal correlation and amplitude in the given sensor. Among the 44 MMR sensors, we took the maximum of *M* (*M*_max_) across the MMR sensors for each method, denoted by $M_{\max}^{DC}$, $M_{\max}^{ICA}$, $M_{\max}^{DC\text{\_}\varphi }$, and $M_{\max}^{ICA\text{\_}\varphi }.\;M_{\max}$ refers to how maximally similar Q components are to the reference standard regarding temporal information when back-projected into the sensor space. Specifically, when one component was selected (*q* = 1), *M*_max_ represented the maximal temporal resemblance to the reference standard when the corresponding component was back-projected into the sensor space. Accordingly, the scatter plot of *C*_max_ and *M*_max_ shows the relationship between the spatial and temporal resemblance to the reference standard in each component.

#### Z-Score and Principal Component Analysis for the Component Distribution Pattern

Two-hundred and four components from each subject should be divided into several groups; MMR-related components (“salient component”) and non-MMR-related components (“inconsequential component”). To classify components, each *M*_max_ and *C*_max_ value derived from all components from all methods (204 × 4 = 816) were individually standardized (i.e., z-scored). Thus, the scatter plot of z-scored *M*_max_ and *C*_max_ reflected the component distribution pattern. For each method (subtraction-*BSS*_*T*/*k*_, subtraction-infomax, weighted-*BSS*_*T*/*k*_, and weighted-infomax), the component locations were classified into four quadrants (left upper [LU]; right upper [RU]; left lower [LL]; and right lower [RL]) by setting the z-score > 1.65 (90%) for both *M*_max_ and *C*_max_, with right referring to high *M*_max_ and upper referring to high *C*_max_. “Salient components” were defined individually in the LU, RU, and RL quadrants. A component in the RU quadrant may be a “major component” with a high contribution to the MMR, whereas a component in the LU quadrant, which has low *M*_max_ and high *C*_max_, is considered a “minor component” of the MMR; most of these components have either small amplitudes or low correlations with the reference standard. A component in the RL quadrant may be a “pseudo-component” regarding the MMR, which suggests that the temporal resemblance is high only in a limited number of MMR sensors. This component may relate to a false (or network) or partial generator of MMRs. A component in the LL quadrant (“inconsequential component”) means irrelevant regarding the MMR or is a component that is related to other ERFs or artifacts.

With successful decomposition, it is expected that only a few components will fall within the RU quadrant, and the rest of the components will fall within the LU, RL, and LL quadrants near the borderlines of coordinate origin. In contrast, unsuccessful decomposition will provide a component distribution pattern where no components fall within the RU quadrant, and all components will fall near the LL quadrant. To investigate the distribution pattern of the salient components, principal component analysis (PCA) was applied to z-scored *M*_max_ and *C*_max_. Two individual PCA components were obtained, with most of the variance being captured by the subspace of the first PCA component (more than 84%; Table 1). The center of the distribution of salient components, taken as the cross-point of the first and second PCA components, and the slope of the first PCA component were obtained.

**Table 1:** Distribution patterns of the salient components.

	**Number of salient components (Z-score > 1.65)**	**Number of salient components (Z-score > 1.96)**	**Z-scored M_**max**_ of the center**	**Z-scored C_**max**_ of the center**	**Slope of the first PCA component**	**Variance of the first PCA component**
Subtraction-*BSS_*T*/*k*_* (± SD)	16.7 ± 2.8 (range, 3–16)	12.9 ± 2.3 (2–9)	3.1 ± 1.4	0.6 ± 0.4	0.29 ± 0.2	0.84 ± 0.07
Weighted-*BSS_*T*/*k*_* (± SD)	8.0 ± 4.4 (13–21)	5.3 ± 2.5 (10–17)	3.7 ± 1.4	1.6 ± 0.5	0.14 ± 0.1	0.94 ± 0.04
Subtraction-infomax (± SD)	36.0 ± 15.3 (16–37)	20.7 ± 8.2 (5–30)	0.8 ± 0.5	1.6 ± 0.7	−0.60 ± 0.4	0.84 ± 0.10
Weighted-infomax (± SD)	26.5 ± 7.5 (16–71)	15.0 ± 7.7 (10–33)	0.7 ± 0.5	1.6 ± 0.6	−0.60 ± 0.4	0.84 ± 0.10

*BSS_T/k_, T/k (fractional) type of decorrelation method; PCA, principal component analysis; SD, standard deviation*.

If a z-score > 1.96 (95%) was set, the number of salient components was small (Table 1), especially in the weighted-*BSS*_*T*/*k*_. PCA seemed unreliable when the input data were <5; thus, a z-score > 1.65 (90%) was applied.

#### The Cumulative Back-Projection of Salient Components

To investigate the contribution of each component to the MMR, components were cumulatively projected back into sensor space (subsection Component Evaluation: Back-Projection and Morphology Similarity), and the spatio-temporal resemblance was compared with the reference standard (subsections Component Evaluation: Cosine Similarity and Component Evaluation: Back-Projection and Morphology Similarity). It is expected that the more components that contribute to the MMR are cumulatively back-projected, the more the back-projected sensors resemble the reference standard. The order of cumulation was determined after sorting by the first PCA component axis (Supplementary Figure 3). Salient components were selected for cumulative back-projection because components below thresholds (inconsequential components in the LL quadrant) are expected to contribute little to the MMR. Corresponding to Eq. (19), *M* was investigated for the cumulative back-projection. The back-projected data in sensor space derived from more than two components have a dynamic topography over time, whereas those derived from one component have a fixed field distribution. Thus, in the cumulative back-projection, *M* was obtained for an average of 44 MMR sensors, not *M*_max_;


(20) $$\begin{array}{l} M_{ave}:=\;mean\left(M\right). \end{array}$$

Thus, *M*_*ave*_ represents both spatial and temporal information regarding the MMR, which reflects the average resemblance to the reference standard. Corresponding to each method, *M*_*ave*_ becomes $M_{ave}^{DC}$, $M_{ave}^{ICA}$, $M_{ave}^{DC\text{\_}\varphi }$, and $M_{ave}^{ICA\text{\_}\;\varphi }$.

#### Relative Contribution

The contribution of a salient component to the MMR or the reference standard is high if a prominent *M*_*ave*_ increment is observed when cumulatively reconstructing one salient component. Thus, the contribution of each component to MMR was defined as


(21) $$\begin{array}{l} \text{Relative contribution }\left(RC\right):\;\frac{M_{ave}\left(c\right)-\;M_{ave}\left(c-1\right)}{M_{ave}\left(q_{all}\right)}\;,\\ \;c=1,\;2,\;\ldots ,\#end. \end{array}$$

where *q*_*all*_ means Q = {1, 2, 3, …, 204}, *c* represents an index number of the salient component according to the sorted order when cumulated (subsection The Cumulative Back-Projection of Salient Components), and #*end* is the index number of the last one. The denominator of Eq. (21) is *M*_*ave*_ when *q* = *q*_*all*_ in Eq. (16), then


(22) $$\begin{array}{l} Z\left(q_{all}\right)=F{\bar{x}}_{\#\text{\_}sub}\left(n,\;q_{all}\right),\;n_{1}\leq n\leq n_{2},\\ \;={\bar{x}}^{Ref}\in {\mathbb{R}}^{L\times \left(n_{2}-n_{1}\right)}. \end{array}$$

Thus, the denominator of Eq. (21) represents *M*_*ave*_ of the reference standard. Corresponding to each method, *RC* becomes *RC*^*DC*^, *RC*^*ICA*^, *RC*^*DC*_φ^, and *RC*^*ICA*_φ^.

As it is expected that *RC* will decrease as *c* increases, we applied the exponential function approximation to the plotted data in the *c-RC* plane;


(23) $$\begin{array}{l} y=\;\beta e^{-\alpha x}, \end{array}$$

where *x* implies *c*, and *y* represents *RC* with coefficients α and β.

### Statistics

To compare the component distribution patterns between the four methods, a two-way repeated-measures analysis of variance (rmANOVA) was used to analyze the center (z-scored *M*_max_ and z-scored *C*_max_, respectively), and slope of the first PCA component with within-subjects factors of APPROACH (subtraction vs. weighted) and DECOMPOSITION (*BSS*_*T*/*k*_ vs. infomax). For the *post hoc* tests, multiple comparisons were performed using paired *t*-tests with Bonferroni correction. The significance level was set at *p* < 0.05.

We counted *c*, where the non-linear approximation reached the 5% threshold. It was assumed that components above the 5% threshold significantly contributed to the MMR and were defined as “dominant components,” whereas those that did not meet the threshold did not contribute to the MMR.

## Results

The analysis comprised four parts (Figure 2, double squares): (i) defining the reference standard based on the spatio-temporal cluster permutation from the sensor-space analysis; (ii) qualitative evaluation of each component based on its similarity to the reference standard; (iii) statistical assessment of component distribution patterns with the z-scored scatter plot; and (iv) the relative contribution of each component.

### Spatio-Temporal Cluster Permutation and Reference Standard

The results of the spatio-temporal cluster permutation are shown in Figure 3A. Among the six clusters, Clusters #1 and #2 (20 left temporal sensors at 96–276 ms with the alpha level of *p* = 0.004, 24 right front-temporal sensors at 105–266 ms with the alpha level of *p* = 0.008) were consistent with the empirical findings. On the other hand, Clusters #3, #4, and #6 contained the late latency and with lower alpha levels of *p* = 0.01. The Cluster #5 was at 102–212 ms mainly from parietal sensors with the alpha level of *p* = 0.008. Therefore, Clusters #1 and #2 were selected to define the reference standard (i.e., a selection of MMR sensors from 44 left temporal and right front-temporal sensors at an MMR time range of 96–276 ms).

Figure 3B represents the grand-averaged ERFs elicited by standard and deviant stimuli and the MMR difference sensor waveforms in sensor space (${\bar{x}}_{sub}$). As indicated by the results of the cluster permutation (Figure 3A topographical map in Clusters #1 and #2), prominent activity occurred in the bilateral temporal and right frontal sensors at the peak latency (Figure 3B topographical map). Note that the offset of the M100 was included in the MMR time for both standard and deviant ERFs (textured arrows in Figure 3B).

### Qualitative Evaluation of Each Component

**Figure 4:**
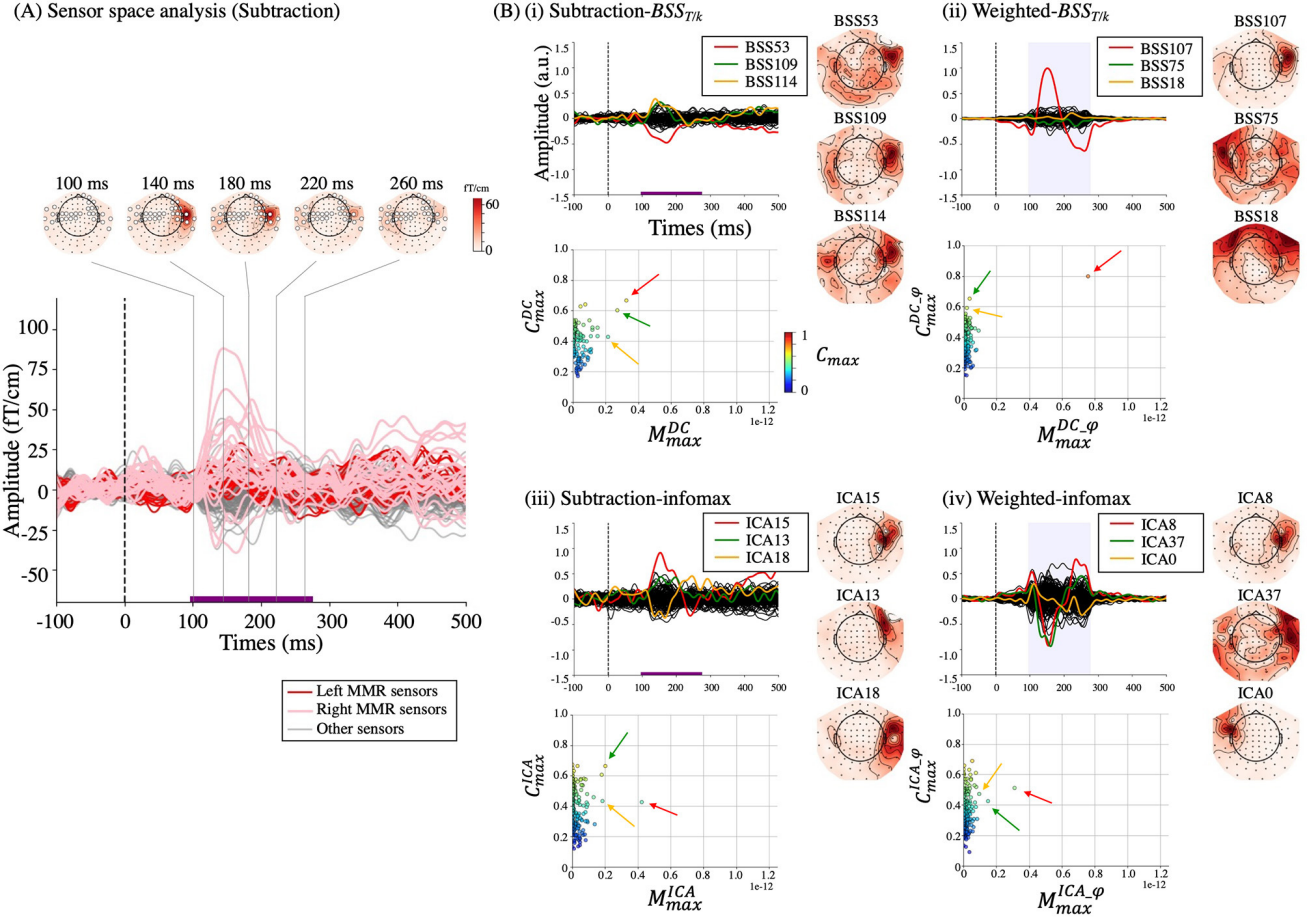
Results from Subject 2. **(A)** Sensor space analysis using subtraction. Red and pink lines from MMR sensors (white circles) within the MMR time (purple line) refer to the reference standard. Decomposition results are shown in **(B)** (i), (ii), (iii), and (iv) for subtraction-*BSS*_*T*/*k*_, weighted-*BSS*_*T*/*k*_, subtraction-infomax, and weighted-infomax, respectively. Upper panels: source waveforms (${\bar{s}}_{sub}\;or\;{\bar{s}}^{\varphi }$), lower panels: scatter plots of *M*_max_ and *C*_max_ for each component. The color map in the scatter plot indicates the value of *C*_max_ (from 0 to 1). In each decomposition result, three components are depicted in different colors (red, green, and yellow) with their corresponding topographical maps. In the scatter plots, the arrows with the same color correspond to the components. These components were selected based on the order of cumulation of the first three components (see subsection The Cumulative Back-Projection of Salient Components). Hereafter, the topographical map takes an arbitrary unit due to matrix *A* in Eq. (2).

**Figure 5:**
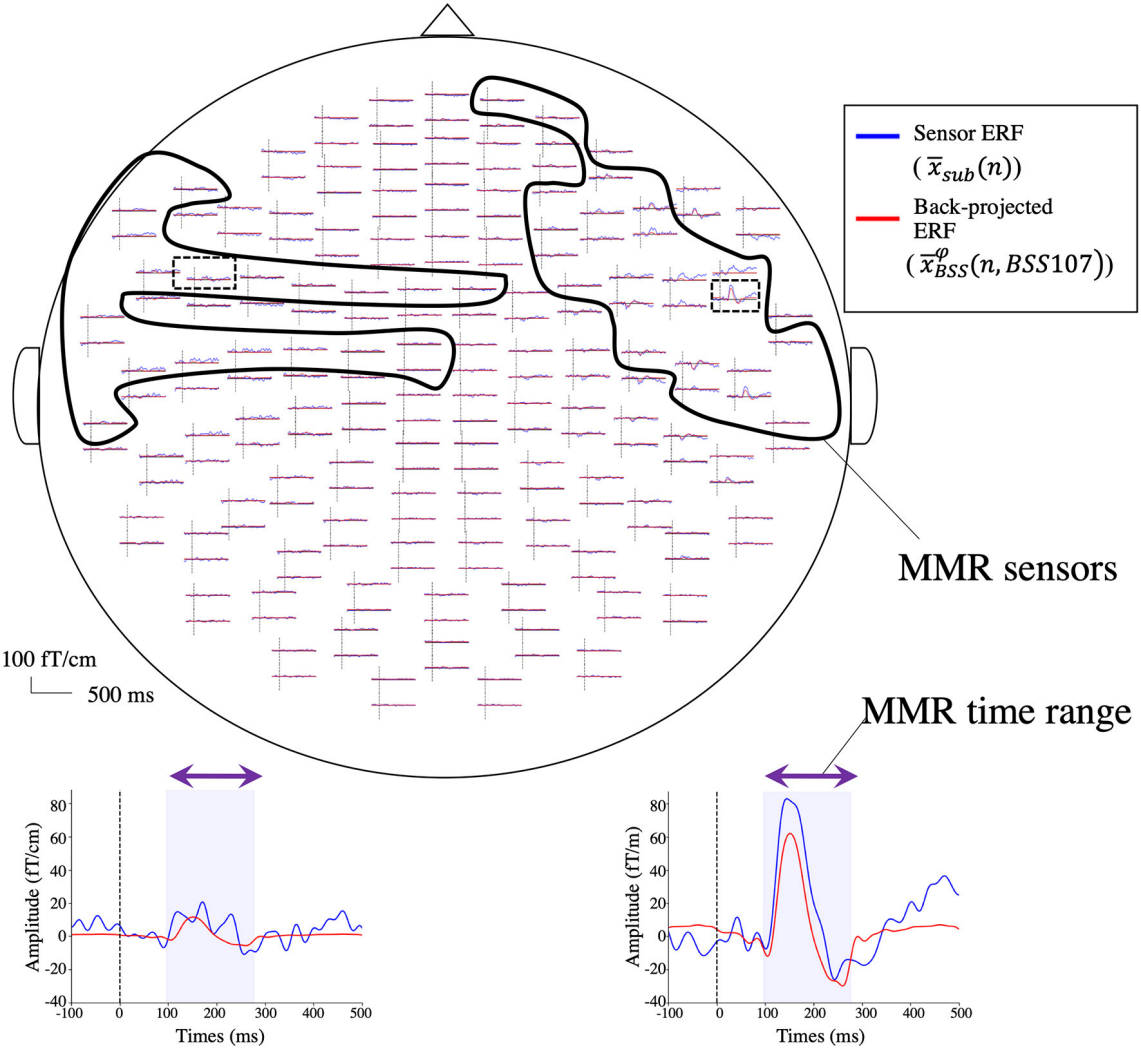
Back-projection of one component (*q* = 1, BSS107) from the weighted-*BSS*_*T*/*k*_ in Subject 2. The blue lines [${\bar{x}}_{sub}\left(n\right)$] indicate the MMR difference sensor waveform obtained using the conventional subtraction approach. The blue lines in the MMR sensors (bold black areas) within the MMR time range (blue shadows) refer to the reference standard [${\bar{x}}^{Ref}\left(n\right)$]. After the back-projection of one component, the ERF was obtained [red lines, ${\bar{x}}_{DC}^{\varphi }\left(n,\;BSS107\right)$]. The maximum of the morphology similarity (*M*_max_) as the temporal resemblance of these two waveforms in each MMR sensor within the MMR time range were investigated. Two representative right and left MMR sensors are shown (dotted area). n, discrete time.

Figure 4 represents the results of the decomposition together with the sensor space analysis of a representative subject (Subject 2). The resemblance of the reference standard (Figure 4A) from this subject was compared with each component from four methods (subtraction-*BSS*_*T*/*k*_, weighted-*BSS*_*T*/*k*_, subtraction-infomax, and weighted-infomax, in Figure 4B(i-iv), respectively). One component in the weighted-*BSS*_*T*/*k*_ was discriminable with similar morphology [Figure 4B(ii) upper panel, red line, BSS107] and topographical map [Figure 4B(ii) right] to the reference standard of the peak time (Figure 4A, 140 ms). When this component was back-projected into the sensor space (Figure 5, red lines), the left and right temporal sensors (dotted areas) within MMR sensors at MMR time range closely represented the reference standard (blue lines). Accordingly, the corresponding component had discriminable $M_{\max}^{DC\text{\_}\varphi }$ and $C_{\max}^{DC\text{\_}\varphi }$ among other components in the scatter plot [Figure 4B(ii) lower panel, red arrow]. Moreover, this component showed a minor additional topographical representation in the left temporal sensors, which corresponded with the reference standard of 260 ms. No components were discriminable using the infomax methods [Figure 4B(iii, iv)]. The subtraction-*BSS*_*T*/*k*_ [Figure 4B(i)] provided two components (red and green arrows) that had a moderate value of $M_{\max}^{DC}$ and $C_{\max}^{DC}$.

**Figure 6:**
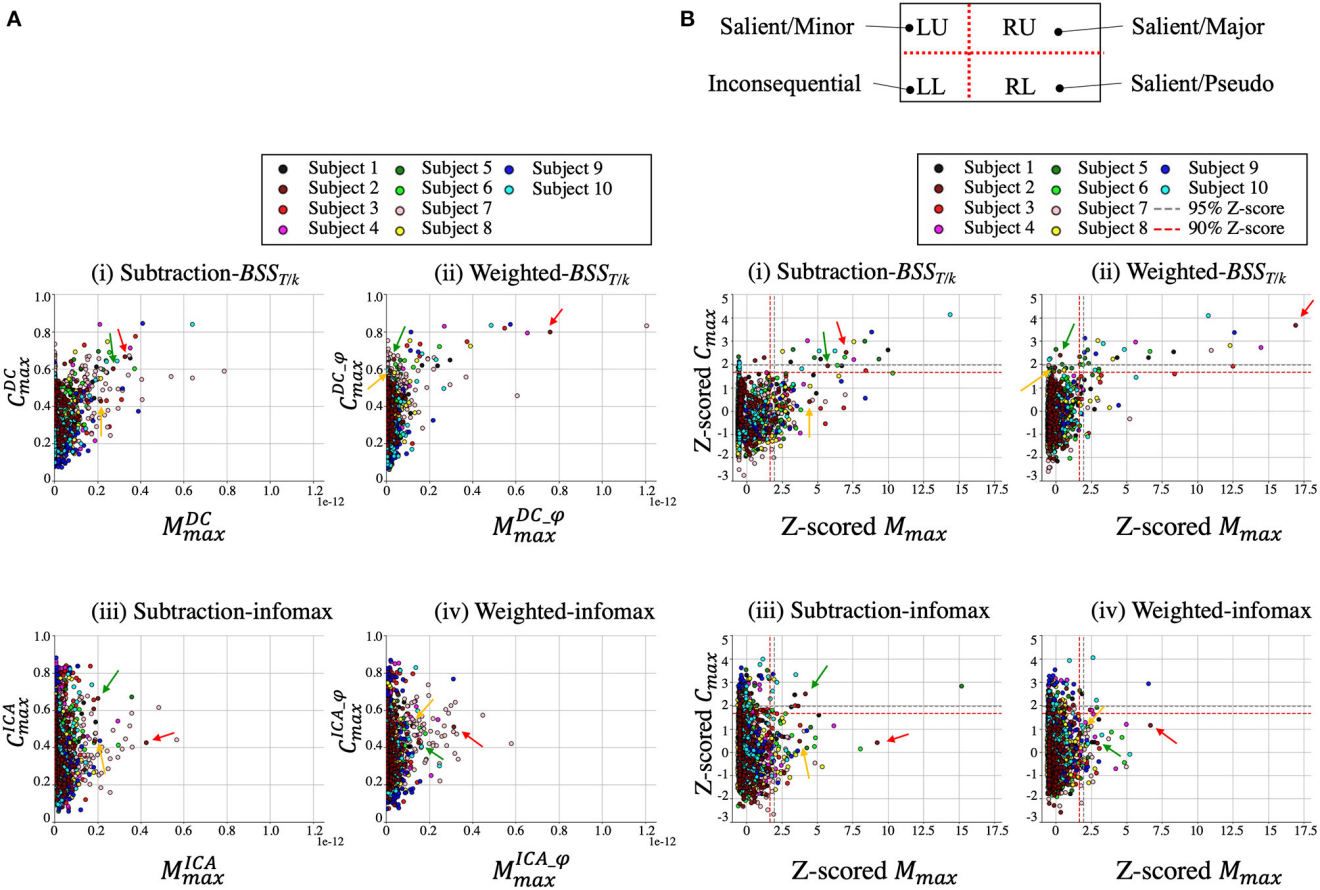
Scatter plots of *M*_max_ and *C*_max_ for each component in the four methods for all subjects **(A)**. Z-scored scatter plots represent the distribution pattern of salient and inconsequential components **(B)**. The four quadrants are divided by red dotted lines (z-score > 1.65 [90%]). Red, green and yellow arrows indicate the corresponding components from Subject 2 in Figure 4.

Scatter plots of *M*_max_ and *C*_max_ for each component are depicted for the four different methods for all subjects (Figure 6A). While most of components had lower *M*_max_ and *C*_max_ values in the four methods, in the weighted-*BSS*_*T*/*k*_ [Figure 6A(ii)], one or a few components represented high $M_{\max}^{DC\text{\_}\varphi }$ and $C_{\max}^{DC\text{\_}\varphi }$ values individually. The z-scored plot shows the distribution of salient (MMR-related) and inconsequential (non-MMR-related) components according to the quadrants based on a 90% z-score (Figure 6B). The salient components were mostly located in the RU quadrant (major) in the weighted-*BSS*_*T*/*k*_ [Figure 6B(ii)], whereas in the subtraction-*BSS*_*T*/*k*_ [Figure 6B(i)], they were equally distributed between the RU (major) and RL (pseudo) quadrants. The two infomax methods [Figure 6B(iii and iv)] had salient components mostly in the LU (minor) or RL (pseudo) quadrants. Most components are inconsequential components in all four methods (the numbers of salient components are shown in Table 1).

### Statistical Assessment of the Component Distribution Pattern

**Figure 7:**
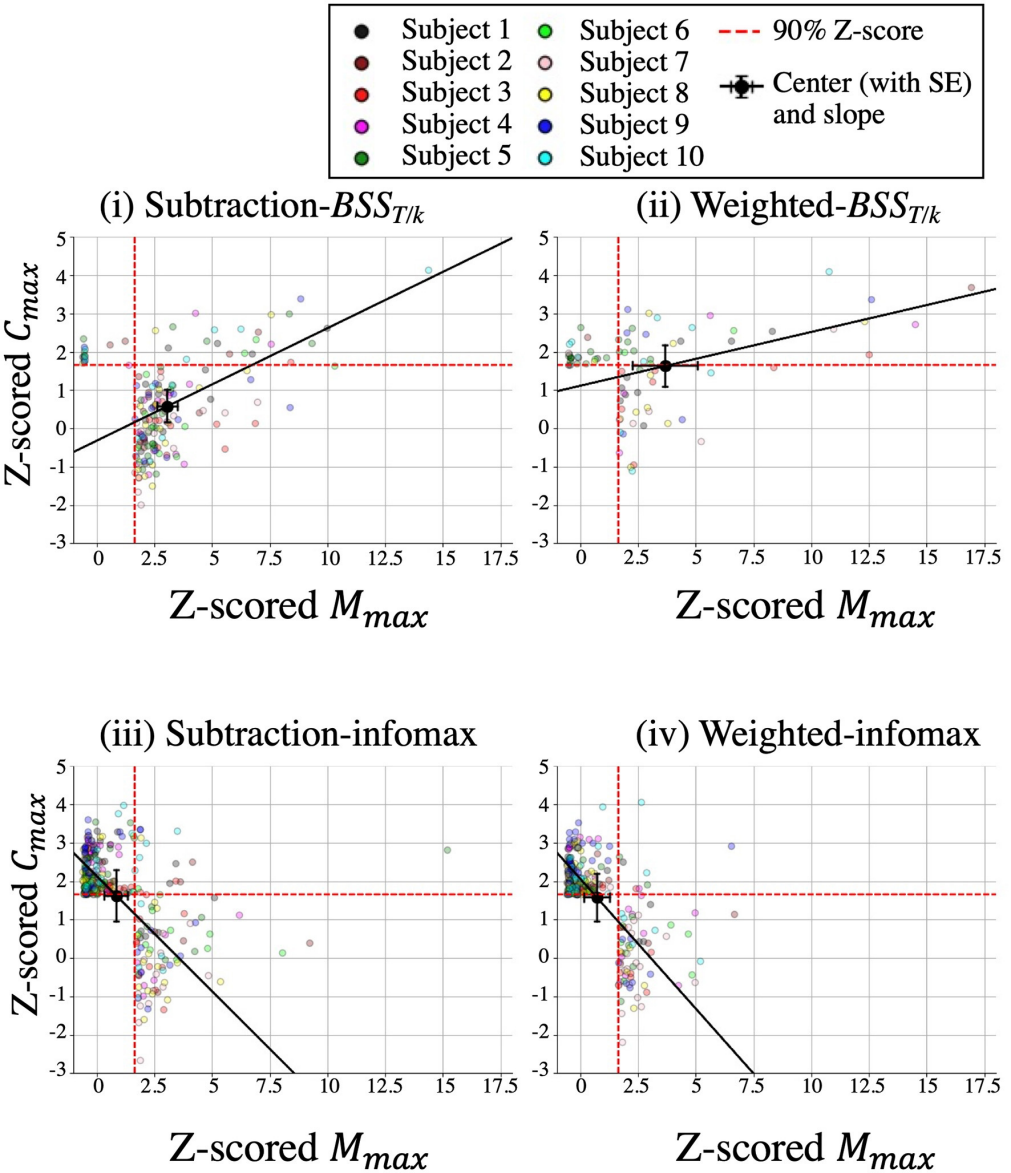
The averaged center of the distribution of the salient components and the slope of the first PCA component superimposed onto the z-scored plots of the salient components. Red dotted lines indicate z-scores > 1.65 (90%). Error bars indicate standard errors (SE).

The component distribution patterns of these salient components (major, minor and pseudo) were further investigated using PCA. The averaged center of the distribution of the salient components and the first PCA component are superimposed on the z-scored plots of salient components in Figure 7 (individual plots are shown in Supplementary Figure 3). The average z-scored *M*_max_, *C*_max_, and slope for the four methods were 3.1 ± 1.4, 0.6 ± 0.4, and 0.29 ± 0.2, respectively, for subtraction-*BSS*_*T*/*k*_ [Figure 7(i)], 3.7 ± 1.4, 1.6 ± 0.5, and 0.14 ± 0.1, respectively, for weighted-*BSS*_*T*/*k*_ [Figure 7(ii)], 0.8 ± 0.5, 1.6 ± 0.7, and −0.60 ± 0.4, respectively, for subtraction-infomax [Figure 7(iii)], and 0.7 ± 0.5, 1.6 ± 0.6, and −0.60 ± 0.4, respectively, for weighted-infomax [Figure 7(iv); Table 1].

**Figure 8:**
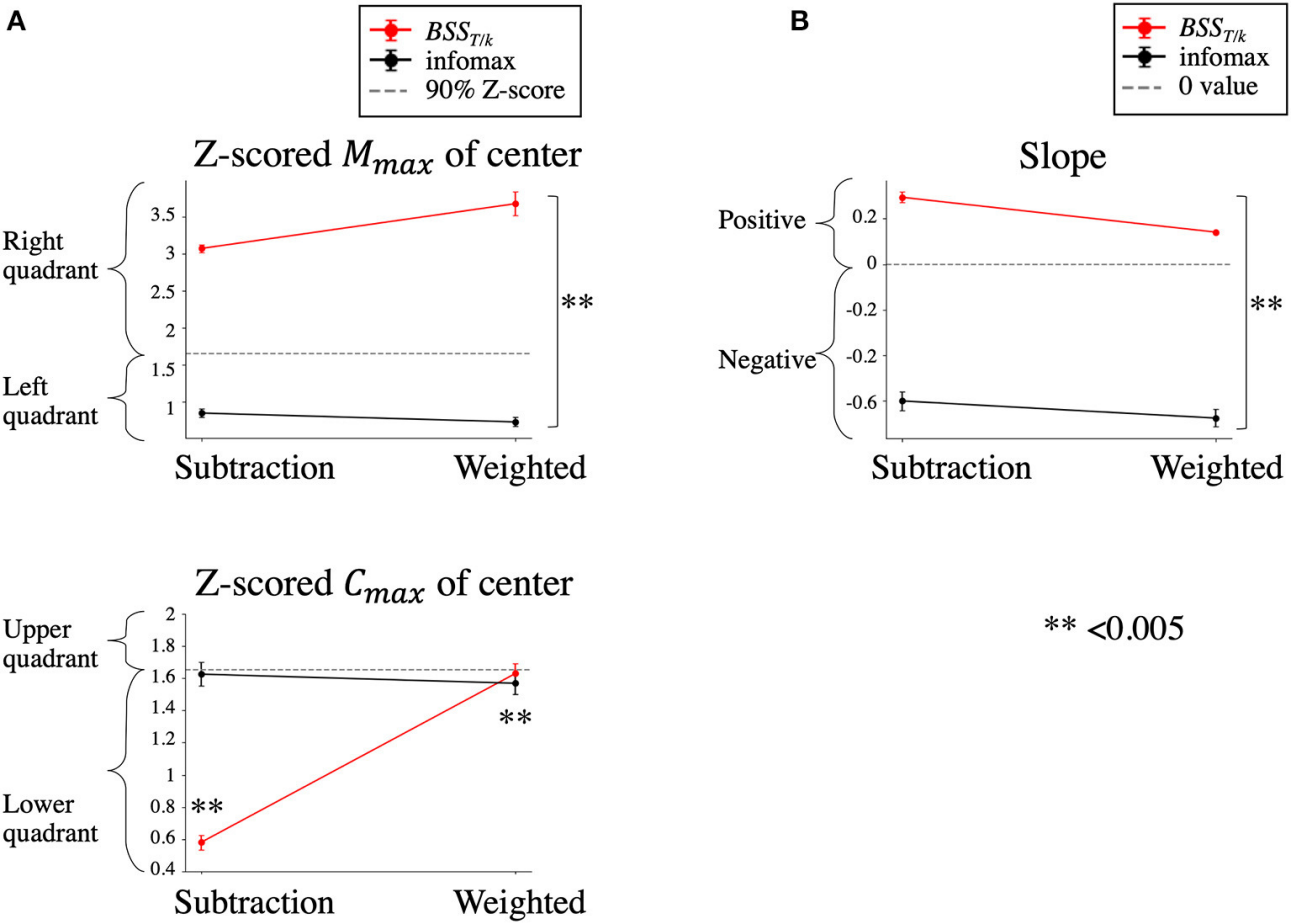
Z-scored *M*_max_ of the center **(A)** (upper panel), z-scored *C*_max_ of the center **(A)** (lower panel), and the slope of the first PCA component **(B)**. Error bars indicate standard errors. See Table 1 for each value. ^**^ < 0.005.

The rmANOVA results of the z-scored *M*_max_ of the center (Figure 8A) revealed a significant main effect of DECOMPOSITION [*F*_(1,9)_ = 76.9, *p* < 0.001], which indicated that the z-scored *M*_max_ in both *BSS*_*T*/*k*_ methods was significantly larger than that in both infomax methods. There was no significant interaction between APPROACH and DECOMPOSITION [*F*_(1,9)_ = 2.7, *p* = 0.1] or main effect of APPROACH [*F*_(1,9)_ = 0.9, *p* = 0.4]. These results suggested that the salient components of both *BSS*_*T*/*k*_ methods were located in the right quadrant, whereas those of both infomax methods were located in the left quadrant.

The rmANOVA results of the z-scored *C*_max_ of the center (Figure 8A) revealed a significant interaction between APPROACH and DECOMPOSITION [*F*_(1,9)_ = 60.4, *p* < 0.001] and significant main effects of APPROACH [*F*_(1,9)_ = 40.2, *p* < 0.001] and DECOMPOSITION [*F*_(1,9)_ = 9.7, *p* < 0.01]. The *post hoc* analysis revealed that the z-scored *C*_max_ of subtraction-*BSS*_*T*/*k*_ was significantly lower than that of weighted-*BSS*_*T*/*k*_ and those of both infomax methods (weighted-*BSS*_*T*/*k*_, *p* < 0.0001; weighted-infomax, *p* < 0.0005; subtraction-infomax, *p* < 0.001). These results suggested that the salient components of the weighted-*BSS*_*T*/*k*_ and both infomax methods were located at the border between the upper and lower quadrants, whereas those of the subtraction-*BSS*_*T*/*k*_ were located in the lower quadrant.

The rmANOVA results of the slope of the first PCA component (Figure 8B) revealed a significant main effect of DECOMPOSITION [*F*_(1,9)_ = 65.5, *p* < 0.001], which indicated that the slope in both *BSS*_*T*/*k*_ methods was significantly larger than that in both infomax methods. There was no significant interaction between APPROACH and DECOMPOSITION [*F*_(1,9)_ = 0.2, *p* = 0.6] and no main effect of APPROACH [*F*_(1,9)_ = 2.4, *p* = 0.2]. These results indicated that the locations of the salient components in both *BSS*_*T*/*k*_ methods had positive spatio-temporal correlations regarding the MMR (i.e., the slope had a positive value), whereas those of both infomax methods had negative correlations (i.e., the slope had a negative value).

In conclusion, the distribution of the salient components was mostly in the RU quadrant (major) with weighted-*BSS*_*T*/*k*_ [Figures 6B(ii), 7(ii)], the RL quadrant (pseudo) with subtraction-*BSS*_*T*/*k*_ [Figures 6B(i), 7(i)], and the LU (minor) or RL (pseudo) quadrants with the two infomax methods [Figures 6B(iii and iv), 7(iii and iv)]. Both *BSS*_*T*/*k*_ methods [Figures 6B(i and ii), 7(i and ii)] showed positive spatio-temporal correlations while both infomax methods showed negative correlations [Figures 6B(iii and iv), 7(iii and iv)].

### The Cumulative Back-Projection and Relative Contribution

**Figure 9:**
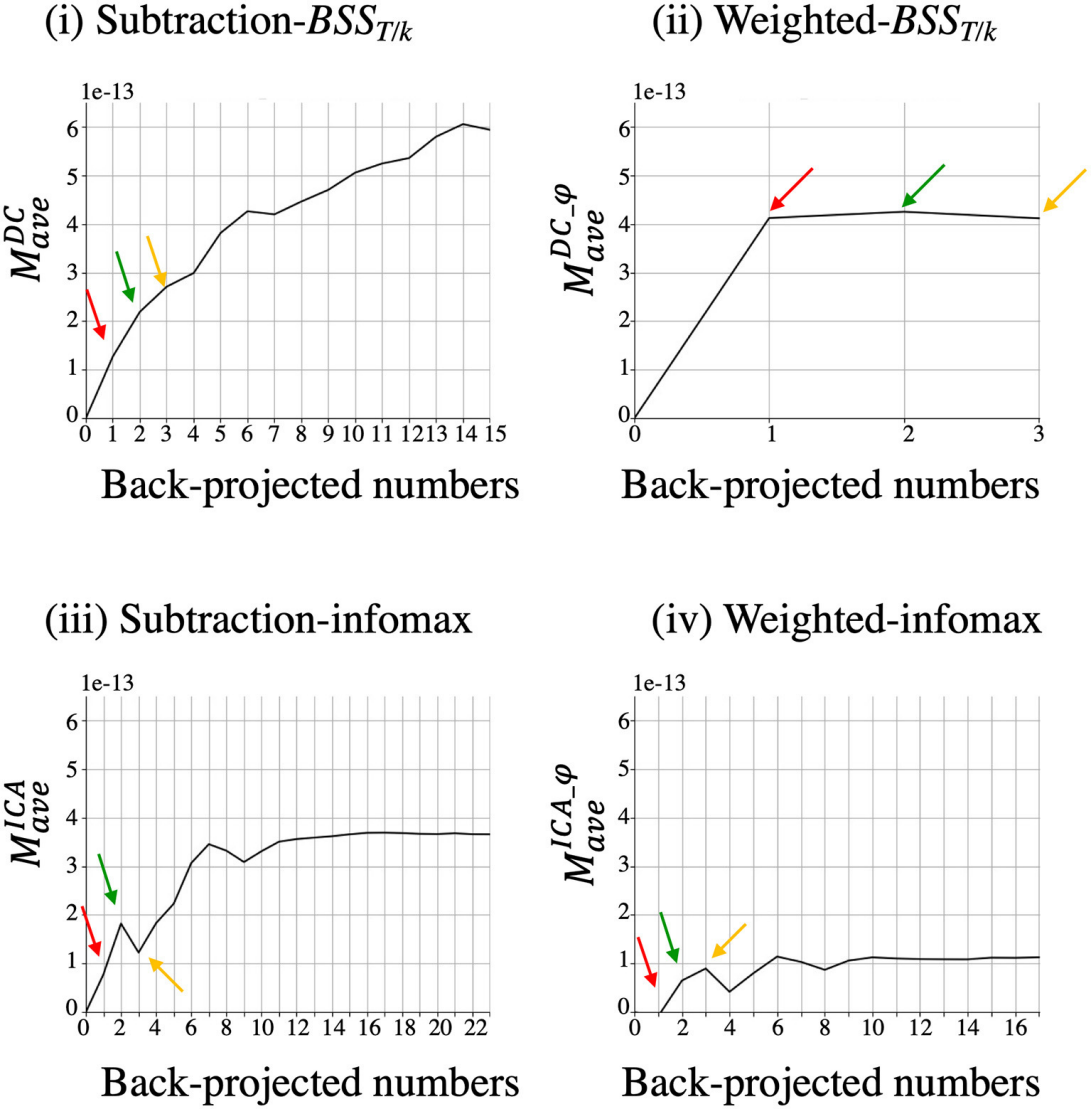
The cumulative back-projection and result of *M*_*ave*_ for the four methods in one subject (Subject 2). The order of cumulation is determined after sorting by the first PCA component axis (Supplementary Figure 3). The number of components reconstructed depended on the number of salient components. Red, green and yellow arrows indicate the corresponding components from Subject 2 in Figure 4.

Figure 9 shows the results of *M*_*ave*_ after cumulative back-projection in a representative subject (Subject 2). The curvature of the weighted-*BSS*_*T*/*k*_ [Figure 9(ii)] was steep in the first component (*c* = 1, red arrow, corresponding to BSS107), which suggested that in the weighted-*BSS*_*T*/*k*_, only one component contributed highly to the MMR. Note that this component was a major component localized on the RU quadrant [Figure 6B(ii)]. On a contrary, other components represent a minimal increase in *M*_*ave*_ [e.g., green and yellow arrows from weighted-*BSS*_*T*/*k*_ in Figure 9(ii) or all three arrows from two infomax methods in Figure 9(iii and iv)]. These were either pseudo- or minor components (Figure 6B). In addition, it is notable that the third component of subtraction-infomax [Figure 9(iii), yellow arrow, corresponding to pseudo-component in Figure 6B(iii)] negatively contributed to the MMR. Moreover, the first component of subtraction-*BSS*_*T*/*k*_ [Figure 9(i), red arrow] showed a mild increment in *M*_*ave*_, which corresponds to this component being classified as a major component in Figure 6B(i).

Supplementary Figure 4 shows the *RC* lines (upper panels) together with their approximate lines (lower panels) in individual subjects. In Subject 2, the approximate lines of the weighted-*BSS*_*T*/*k*_ show that the first component (red arrow) represented a contribution as high as 30%, whereas later components (green and yellow arrows) provided much lower contributions. We counted *c*, where the non-linear approximation reached the 5% threshold (gray dotted lines; i.e., the dominant components). The number of dominant components is shown in Table 2. In the weighted-*BSS*_*T*/*k*_, 1–3 components significantly contributed to the MMR, except for one subject (Subject 5). In the subtraction-*BSS*_*T*/*k*_, 2–6 components contributed to the MMR. The two infomax methods had few components that significantly contributed to the MMR. These results indicated that one or a few dominant components contributed to the MMR in weighted-*BSS*_*T*/*k*_, whereas no components represented the MMR in infomax.

**Table 2:** Numbers of dominant components.

**Subject No**.	**Subtraction-*BSS_***T*/*k***_***	**Weighted-*BSS_***T*/*k***_***	**Subtraction-infomax**	**Weighted-infomax**
Subject 1	5	2	None	None
Subject 2	3	1	1	None
Subject 3	6	3	None	None
Subject 4	2	2	None	1
Subject 5	3	None	None	None
Subject 6	3	1	2	None
Subject 7	4	2	None	None
Subject 8	5	3	None	None
Subject 9	4	1	None	1
Subject 10	4	3	None	None

*BSS_T/k_, T/k (fractional) type of decorrelation method*.

## Discussion

In the current multi-channel MEG study, we demonstrated that our novel weighted-*BSS*_*T*/k_ method using only deviant epochs (deviant concatenation) could extract an MMR confined to one or a few dominant components (Figures 4, 6, 9, Supplementary Figure 4, and Table 2). In the subtraction-*BSS*_*T*/k_/weighted-*BSS*_*T*/k_, the salient components showed positive spatio-temporal correlations with the MMR (Figures 7, 8, and Supplementary Figure 3). However, ICA decomposed the MMR into an assembly of minor or pseudo components with negative spatio-temporal correlations. Specifically, our method avoids having to use the conventional subtraction approach to reveal the MMR. Our method may help with the use of the MMR in basic and clinical research.

### The Conventional Subtraction Approach to Reveal the MMR

The MMR has been widely used in many fields of human neuroscience (10, 15, 16). Conventionally, the subtraction approach was needed to extract the MMR from other auditory ERP/ERF. However, there are several problems with such a method, which include increased noise and the inability to exclude neural adaptation. Several approaches have been proposed to avoid the neural adaptation problem (53, 57); however, all such approaches depend on subtraction. Our novel approach avoids subtraction. In general, the MMR is a relative component because a common response is included in standard and deviant ERFs, and the MMR is then defined as the difference waveform based on the original theory underlying the MMR (i.e., the memory-comparison process). The MMR should be present in deviant epochs but not in standard epochs. Thus, only deviant epochs are needed for its decomposition.

### Periodical Arrangements and Weight Assignments

We made two assumptions underlying the successful decomposition of the weighted-*BSS*_*T*/*k*_: (1) The MMR occurs periodically within a specific time range (i.e., the MMR time range) and in the deviant epochs; (2) Exogenous/obligatory ERFs highly correlate with themselves in the non-MMR time. Originally, *BSS*_*T*/*k*_ was expected to highlight periodic signals using *T* (35–38). The MMR time range (96–276 ms) was defined according to the spatio-temporal cluster permutation analysis, which was assumed to reveal the statistically significant time range in which the MMR occurs. Both the offset response of the M100 and the MMR fall into this time range, whereas the onset response of the M100 occurs outside of the time range (Figure 3B). Assigning a weight to this time range may minimize the joint M100 and MMR effect. The weighting emphasizes the target response (i.e., the MMR) within the window, whereas the response outside the window (i.e., the onset of the M100) takes away the response (i.e., the offset of the M100) if they are highly correlated. Analogous to the subtraction approach (as subtraction separates such responses by subtracting deviant responses from standard responses), the weight assignment on a specific time range may differentiate the MMR from other responses. Supplementary Data (2-1) and Supplementary Figure 5 support our assumptions; the assignment of the weight outside the M100 in the standard epochs did not result in the extraction of remarkable components that represent the M100 (Supplementary Figure 5B).

### Significance of our Approach

We obtained four main findings. First, the weighted-*BSS*_*T*/*k*_ decomposed one or a few components (<3) that manifested the MMR among the many components obtained from multi-channel data (Figures 4, 6, 9, Supplementary Figure 4, and Table 2). We refer to this decomposition result as specification. Multi-channel recordings of electromagnetic fields emerging from neural currents in the brain generate large amounts of data (28). Thus, this specification makes interpretation and comparisons among groups easier. Our primary aim was to extract MMR in a few dominant components. The dominant component was the component that had the most discriminable *M*_max_ and *C*_max_, and thus, it contributed most highly to the MMR (Figure 9, Supplementary Figure 4, and Table 2). We do not assume that the dominant component manifests a single MMR source; instead, it may represent the network or a series of MMR sources (Figure 4). Other irrelevant activities were redistributed among the remaining components. Since our method (*BSS*_*T*/*k*_) depends on the theory that utilized correlations between components instead of strong independence (i.e., ICA), it would result in extracting components with keeping physiological correlation that may represent several generators or network of MMR. If bitemporal and frontal MMR sources are highly correlated, with a certain delay, these sources should be extracted in a few components using our time-delayed correlation method. Indeed, it is known that these sources have separate temporal dynamics (58) but interact with each other (59). In contrast, it is difficult to identify any dominant components using ICA, where each extracted component represents one or two dipolar sources. This is discussed in the following section.

Second, the decomposed components revealed positive spatio-temporal correlations regarding the MMR, and the center of the distribution of the salient components was in the RU (major) quadrant (Figures 6–8, Supplementary Figure 3, and Table 1). According to Eq. (2), the decomposed component contains the mixing matrix (spatial) and signal source (temporal). A positive spatio-temporal correlation in the decomposed component suggests that the component is physiologically meaningful (9). In turn, with a positive spatio-temporal correlation, a component that shows the most similar morphology regarding the MMR also has the most similar topography regarding the MMR. This relationship is particularly important when targeting the response with an unknown generator source. The temporal information can be mutually applicable to the detection of the target, without a priori knowledge of its precise generator. For example, in Figure 4B(ii), if the MMR topography is unknown, BSS107 can be selected as the MMR component based on its discriminable amplitude.

Third, each component was obtained from individual data and the results were statistically significant. This indicated that weighted-*BSS*_*T*/*k*_ is generally applicable to individual subjects, unlike group-ICA.

Fourth, a new cohort from subjects with low SNR in the sensor-space analysis regarding MMR (subsection Spatio-Temporal Cluster Permutation to Define the MMR Time Ranges and Sensors or the Reference Standard) demonstrated a few MMR-related components in weighted-*BSS*_*T*/*k*_ when the same MMR time range was used for the weight assignment [Supplementary Data (2-2) and Supplementary Figure 6]. This MMR time range was independently determined in this cohort. These results may indicate that the generous setting of the weight time range can be available as long as the crucial time range is covered.

Based on these results, the application of our approach provides potential benefits that the sensor-space subtraction method does not, despite its status as the current gold standard for revealing MMR. Our single-trial, contrast-free approach would minimize the effect of refractoriness and maximize the temporal information underlying the neural mechanism of MMR. Our approach would therefore provide a new approach toward investigating further insights into the physiology of MMR.

### Comparison With ICA

Both ICA methods (subtraction- and weighted-infomax) consisted of a collection of minor or pseudo components (Figures 4, 6, 9, Supplementary Figure 4, and Table 2). Most components were located in the LU (minor) or RL (pseudo) quadrants (Figures 6–8 and Table 1). The slope of the first PCA component showed a negative spatio-temporal correlation (Figures 7, 8, Supplementary Figure 3, and Table 1). There were no dominant components that manifested the MMR in either of the ICA methods (Table 2). The decomposition method in ICA is based on stochastic properties and does not depend on the time structure; thus, spatio-temporal dissociations may occur (34). Several papers have reported successful decomposition of the MMR using ICA (9, 25, 60–64); however, most results were derived from oligo-channel recordings. When the number of sensors/channels sensing the MMR is relatively small, the MMR can be extracted by one or a few components. However, such specification in multi-channel data is rarely shown in ICA studies because a greater number of channels results in poorer estimation accuracy of the components (25). If we assume fewer numbers of sources (e.g., tens) but use larger numbers (204) of sensors for ICA decomposition, the components of interest will likely be (i) split into sub-components and (ii) located where the SNR of each component is reduced. This is consistent with our previous work where ICA decomposition showed fragments of interictal epileptiform discharges from a single epileptogenic zone (42). Furthermore, most ICA studies are based on cluster analysis (e.g., group-ICA), not individual analysis. Generalization of the application of ICA to the MMR was not demonstrated in these studies.

Lastly, although subtraction-*BSS*_*T*/k_ follows the conventional subtraction approach, it performed better than the two ICA methods, especially the subtraction-infomax. The center of the distribution of the salient components was in the RL quadrant (pseudo), yet it maintained a positive spatio-temporal correlation (Figures 6–8, Supplementary Figure 3, and Table 1). A possible interpretation of these findings is that these components may represent the partial generators of MMR sensors. The difference between subtraction-*BSS*_*T*/k_ and subtraction-infomax may explain the theoretical difference between *BSS*_*T*/k_ and infomax (time-delayed correlation vs. strong independence). The decomposition of the subtraction-*BSS*_*T*/k_ was less successful than that of the weighted-*BSS*_*T*/k_. There were more dominant components (<6; Table 2) in the subtraction-*BSS*_*T*/k_ than there were in the weighted-*BSS*_*T*/k_. From the viewpoint of specification, fewer dominant components are desired. In conclusion, both *BSS*_*T*/k_ methods, which use time structure, performed well in extracting the MMR; however, the weighted approach was the most sensitive.

### Future Perspectives

The current study aimed to extract the MMR as a distinct component using a combination of the periodical arrangement and assignment of a weight. The specific effect of each technique should be investigated in a future study, which may help achieve a better understanding of the physiology of the MMR.

Because there was no confidence in terms of source localization of extracted components, although there are several ICA and SOBI studies (9, 32, 41), this view may provide potential benefits given that components may encompass several sources or networks of MMR. This should be investigated in future studies.

Our method is not dependent on the number of components. Our motivation was not to apply dimension reduction to maximize the multi-channel MEG data. However, the application of our method to different numbers of sensors, different MEG systems, or another type of sensor (magnetometer) is an interesting but open question. Theoretically, our weighted method can possibly be applied to any clinical neurophysiology data to investigate ERFs, which include higher cognitive functions where the elicitation of the target requires subtraction, and the target is subject to a specific assumption about the time window in which it occurs in multi-channel data. In a paradigm where stimuli are jittered and thus are not periodic, our weighted method will also be applicable by concatenating the epochs.

## Limitations

There are several methodological concerns to our study: (i) The spatio-temporal cluster permutation provided several clusters (Figure 3A); however, we did not select all of these. We selected the most reliable clusters that covered 100–250 ms and the bitemporal sensors (7, 17) since the vast majority of EEG studies of MMR generators confirmed these; however, the parietal generator in the later latency (e.g., Figure 3A Clusters #5 and #6) was suggested in several studies (9, 10) and should be investigated in a future study. (ii) The SOA of the current study was relatively short so that the brain response could return to the baseline. This short time range may have concatenation artifacts when deviant concatenation. However, in the weighted-*BSS*_*T*/*k*_ method, the amplitude outside of the window was 0.2 (Eq. 9). Therefore, concatenation artifacts, if any, should be limited. (iii) The window function was set as a rectangular window, which may cause a tingling effect. The selection of a window function should be based on a hypothesis; in the current study, we assumed that the crucial time range of MMR is equally distributed at 96–276 ms based on our data-driven approach, even though this time range is not assumed to have a unique significance. However, the non-rectangular window can be used according to the hypothesis. Therefore, we uploaded the source code of weighted-*BSS*_*T*/*k*_ to GitHub (https://github.com/fractionalTypeBSS/BSSTk.git) to enable users to apply it according to their hypothesis and select so that users can use it based on their hypothesis to choose the window function and time range. (iv) The sample size was relatively small for fully describing the performance of our new approach. However, generalization, as well as the validity of our approach, is supported by our additional analysis in a separate cohort [Supplementary Data (2-2) and Supplementary Figure 6].

## Conclusions

We proposed a novel weighted method for extracting the MMR from multi-channel MEG data. Compared with ICA, our weighted-*BSS*_*T*/*k*_ method was more sensitive in highlighting the MMR in one or a few dominant components with positive spatio-temporal correlations. This new approach which used only deviant epochs could replace or complement the conventional subtraction approach. Our method may facilitate the use of the MMR in basic and clinical research and provide a novel approach to analyze complex event-related MEG and EEG data.

## Data Availability Statement

The original contributions presented in the study are included in the article/Supplementary Material, further inquiries can be directed to the corresponding author.

## Ethics Statement

The studies involving human participants were reviewed and approved by the Ethics Committee of Kyushu University. The patients/participants provided their written informed consent to participate in this study.

## Author Contributions

TMat, SS, JA, and KK: study conception and design. TMat, TMae, and ST: data collection. TMat and KK: analysis and interpretation of results. TMat: draft manuscript preparation. YG, SK, and MH: revision of manuscript. All authors approved the final version of the manuscript.

## Funding

This work was supported by JSPS KAKENHI Grant No. JP20J00552; Nakatani Foundation for Advancement of Measuring Technologies in Biomedical Engineering; the Japan Epilepsy Research Foundation; the Osaka Medical Research Foundation for Intractable Diseases; and the National Institutes of Health [Grants Nos. 5R01NS104585, R01DC016915, R01DC016765, and R01DC017991].

## Conflict of Interest

The authors declare that the research was conducted in the absence of any commercial or financial relationships that could be construed as a potential conflict of interest.

## Publisher's Note

All claims expressed in this article are solely those of the authors and do not necessarily represent those of their affiliated organizations, or those of the publisher, the editors and the reviewers. Any product that may be evaluated in this article, or claim that may be made by its manufacturer, is not guaranteed or endorsed by the publisher.

## Abbreviations

MMR: mismatch response
EEG: electroencephalography
MEG: magnetoencephalography
DC: decorrelation method
ERP: event-related potential
ERF: event-related field
BSS: blind source separation
SNR: signal-to-noise ratio
ICA: independent component analysis
SOBI: second-order blind identification
*BSS*_*T*/*k*_: *T/k* (fractional) type of decorrelation method
SOA: stimulus onset asynchrony
TSSS: temporal signal space separation method
LU: left upper
RU: right upper
LL: left lower
RL: right lower
PCA: principal component analysis
rmANOVA: repeated-measures analysis of variance.
