# Supplementary figures and images for: Weighted Blind Source Separation Can Decompose the Frequency Mismatch Response by Deviant Concatenation: An MEG Study
Source: Front Neurol. 2022 Feb 25;13:762497. doi: 10.3389/fneur.2022.762497 (PMC8916481; doi:10.3389/fneur.2022.762497)

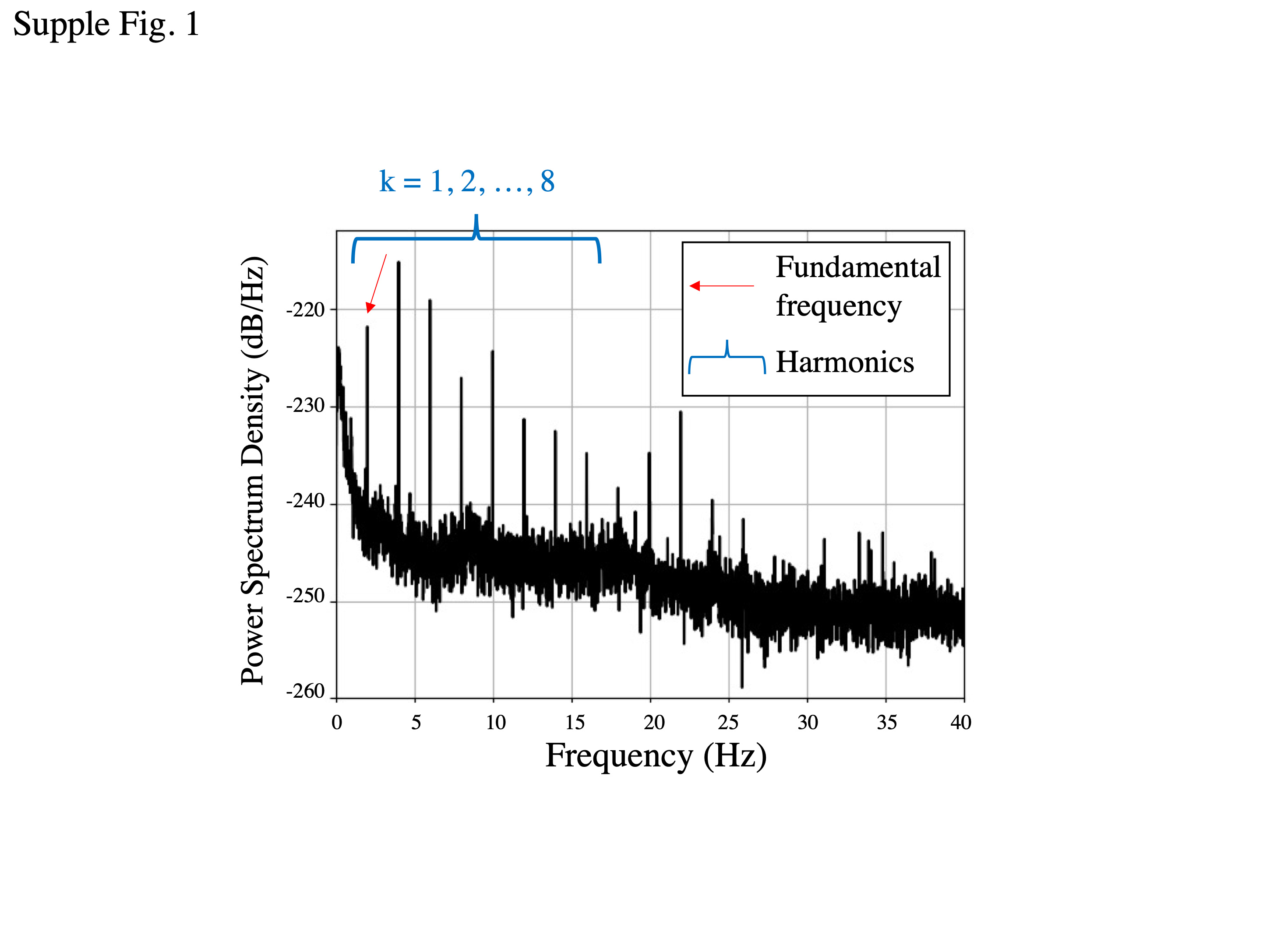

Supplement: Supplementary file 1 [file Image_1.TIFF]

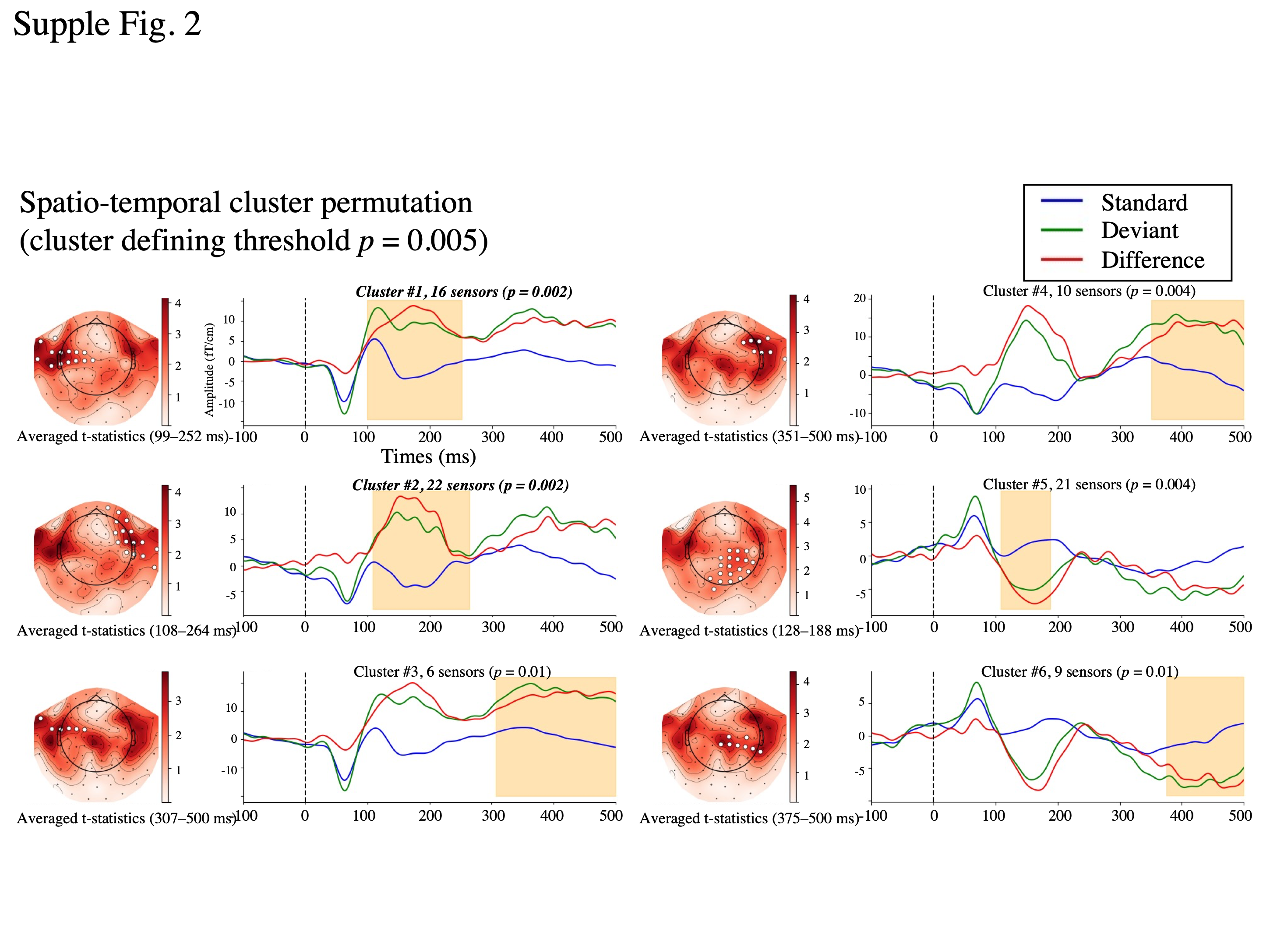

Supplement: Supplementary file 2 [file Image_2.TIFF]

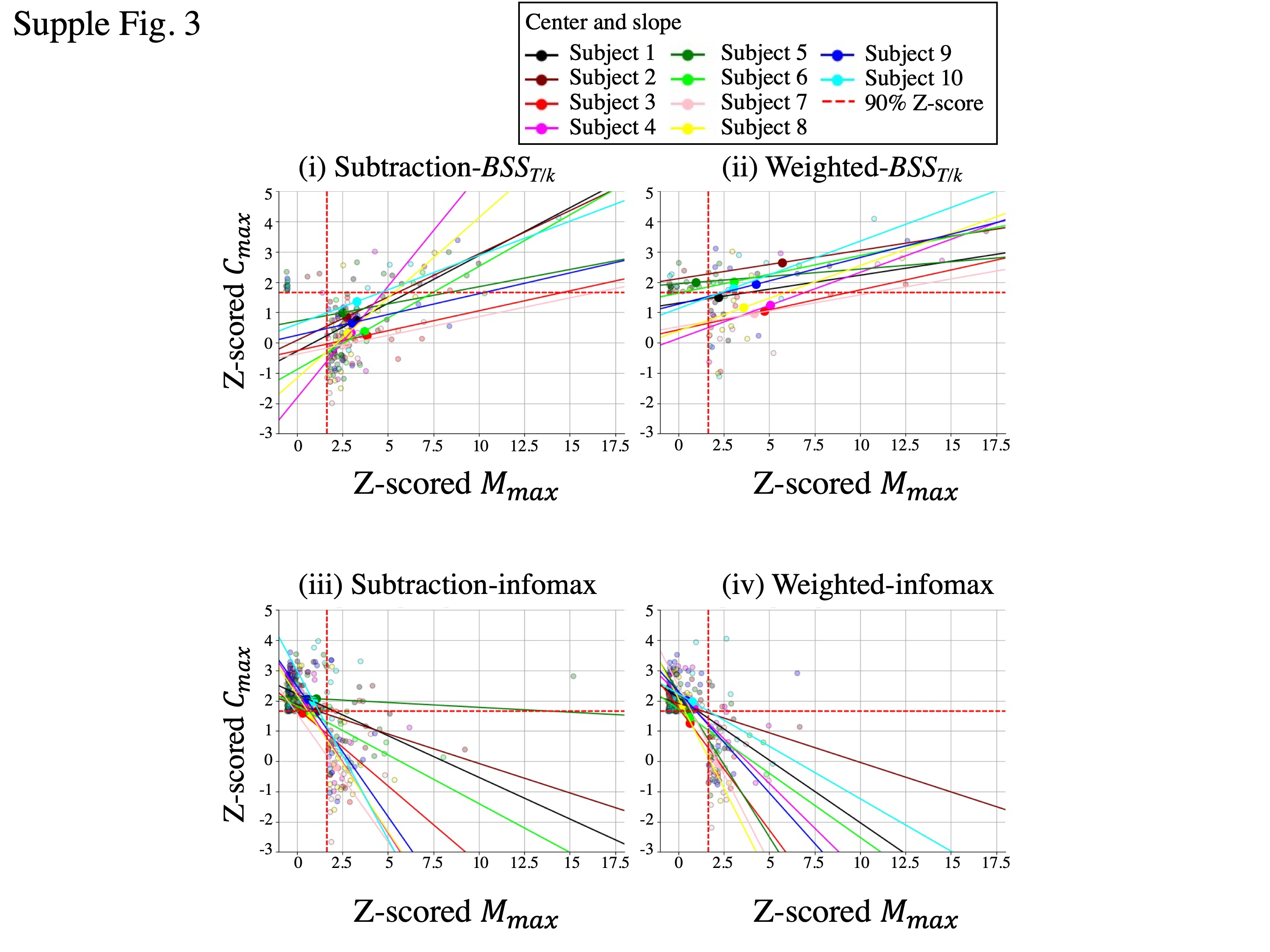

Supplement: Supplementary file 3 [file Image_3.TIFF]

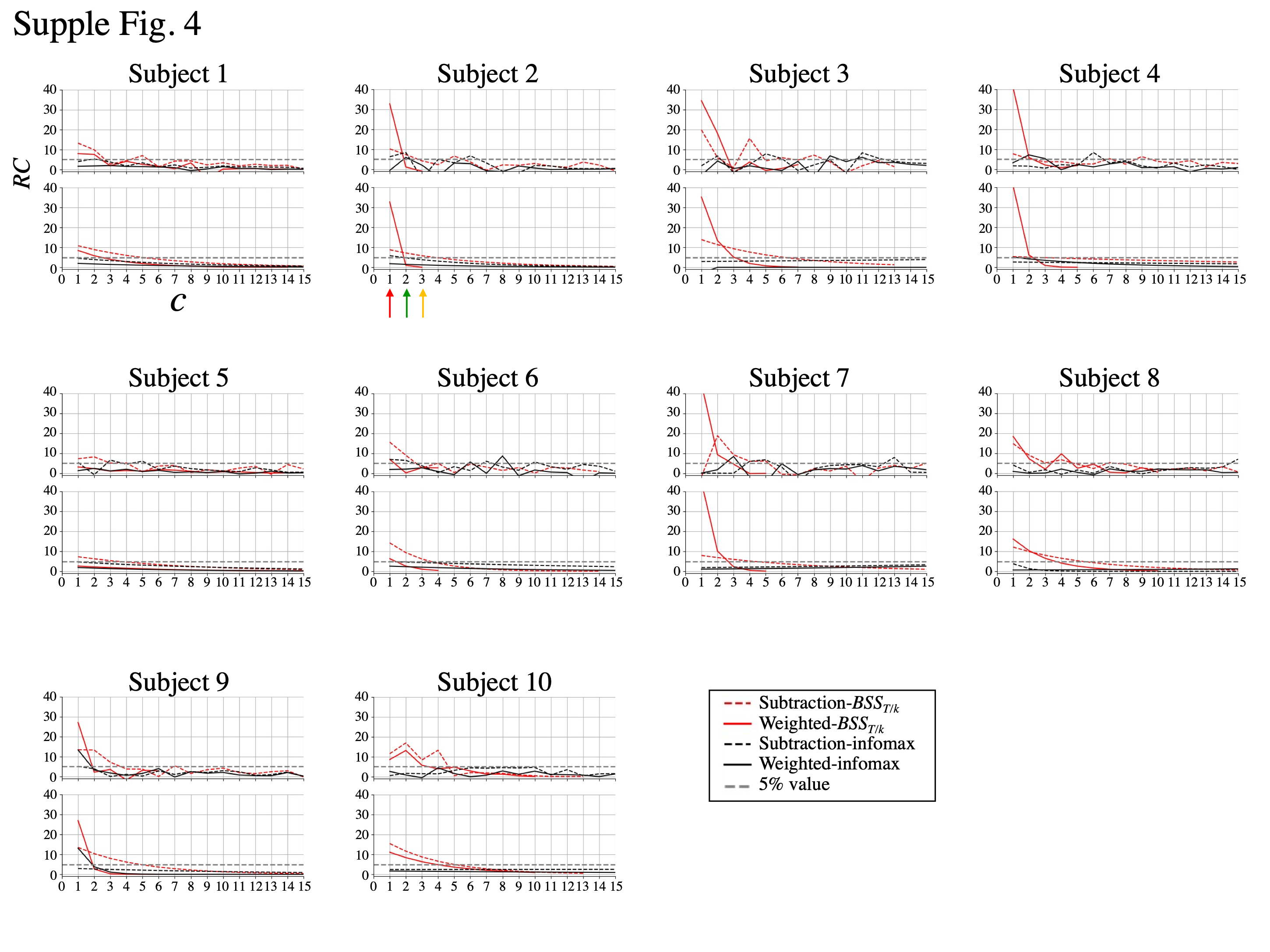

Supplement: Supplementary file 4 [file Image_4.TIFF]

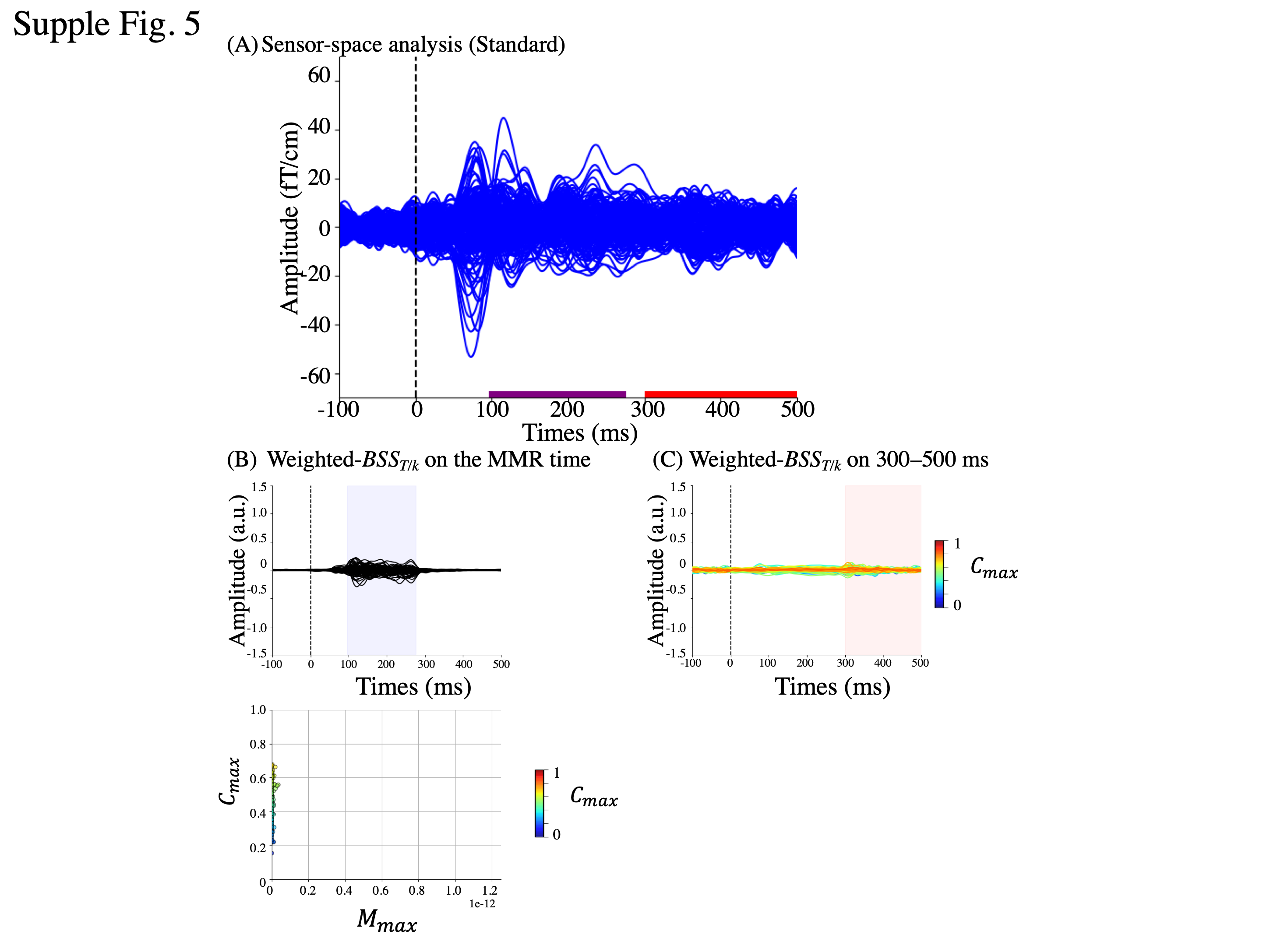

Supplement: Supplementary file 5 [file Image_5.TIFF]

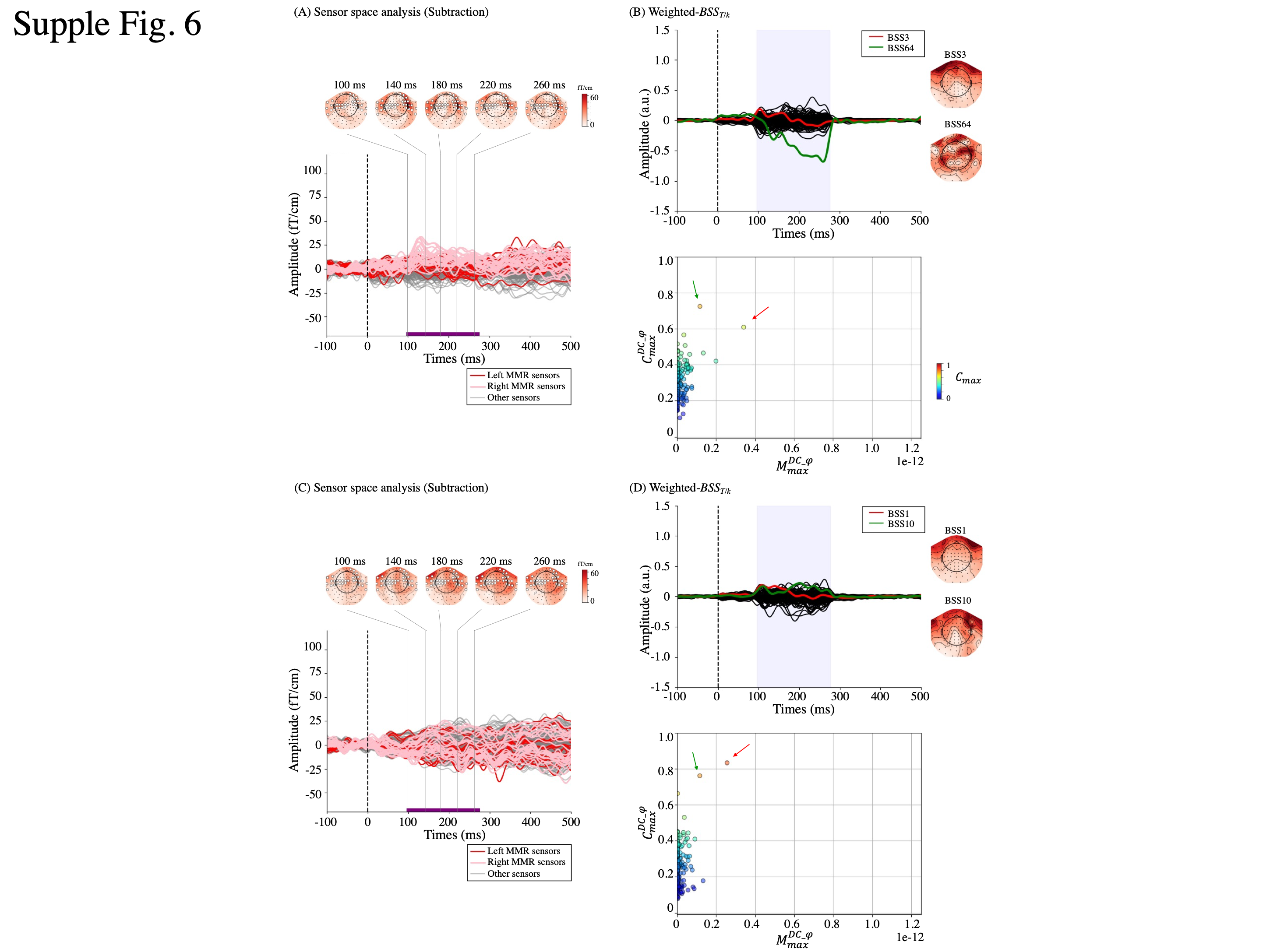

Supplement: Supplementary file 6 [file Image_6.TIFF]
