# Supplementary material for: Weighted Blind Source Separation Can Decompose the Frequency Mismatch Response by Deviant Concatenation: An MEG Study
Source: Front Neurol. 2022 Feb 25;13:762497. doi: 10.3389/fneur.2022.762497 (PMC8916481; doi:10.3389/fneur.2022.762497)
Supplement: Supplementary file 7 [file Data_Sheet_1.docx]

**Supplementary data**

**(1) Setting of parameters; *T* and *k* (Supplementary Fig. 1)**

According to the Fourier series, 2 Hz (*f_p_ = 1/ T*) periodic signals have line peaks with a fundamental and its harmonics (2, 4, 6...,2*k, Hz) in power spectrum density (PSD). In turn, the frequency spectrum represented by line peaks will contain features of the waveform (time-series data, i.e., MMR). Therefore, the ideal parameter of *k* to represent MMR should be infinite (k = ∞); however, if infinity is selected, the decomposition results (*BSS_T/k_* or SOBI) would fall into strong independence, which is equivalent to ICA (see introduction). The number of harmonics (*k*) we should take can be arbitrary; however, it can reasonably be determined in the context of noise. This has been shown in a previous paper (Kishida K. Phys Rev E Stat Nonlin Soft Matter Phys, 2009).

We used data from a representative subject (Subject 2). The MMR difference waveform, which was calculated by subtracting the averaged deviant ERFs from the averaged standard ERF, was used with its 500-ms duration (0–500 ms), which corresponded to a rate of 2-Hz. The representative sensor from the right temporal region (MEG1442) was obtained from the MMR difference waveform (see Fig. 5A). Because the duration of 500 ms was too short to obtain the PSD, we elongated using concatenation to form 512 s of data. Then, brain noises from one occipital sensor (MEG2142) were added to the data. Finally, we obtained the PSD of noise-contained repetitive MMR data, which ideally represented the characteristics of the MMR in the frequency domain. Supplementary Fig. 2 shows the fundamental frequency and its harmonics in 2 Hz intervals in a mixture of brain noise with 1/f and an alpha peak. This data-driven approach reasonably signified 16 Hz (*k* = 8) as effective in covering the harmonics above noise.

**(2) Validity of the weighted approach**

To investigate the validity of the weighted approach, two further analyses were conducted; i) using weighted standard epochs from one random subject, and ii) using a new cohort from two subjects previously excluded because of low SNR.

**(2-1) Weighted standard epochs**

We randomly selected one subject (Subject 7) and weighted 96–276 ms of the standard epochs (Supplementary Fig. 5A purple line) or 300–500 ms (red line). The former time range was the same as the MMR time, whereas the latter was a time range in which we expected no significant activity to occur. We hypothesized that the application of weighted-*BSS_T/k_* to these standard epochs would result in no MMR-related components being extracted from the former time range and no significant components being shown in the latter. Using weighted standard epochs, several steps were followed to make the ERF. For the former time range, $M_{max}$ and $C_{max}$ were obtained using the same spatio-temporal definition as that used for the MMR (i.e., resemblance to the reference standard). For the latter time range (300–500 ms), a corresponding definition could not be determined; thus, $C_{max}$ was simply obtained within this time range.

The ERF obtained from the weighted-*BSS_T/k_* on the MMR time is shown in the upper panel in Supplementary Fig. 5B. No prominent component was shown in the scatter plot of $M_{max}$ and $C_{max}$ (Supplementary Fig. 5B, lower panel). This result indicated that no MMR-related components were extracted in this time range using the weighted standard epochs. In addition, no remarkable components that represented the M100 were extracted in the non-MMR time. An ERF with a weight in the 300–500 ms time range is shown in Supplementary Fig. 5C. There were no outstanding components. This result indicated that no significant components were extracted within or outside this time range.

**(2-2) Weighted-*BSS_T/k_* in a new cohort from two subjects with low SNR**

Two subjects were included in this analysis who had been excluded (subsection 2.4.5) because of low SNR in the sensor-space analysis. We applied the same MMR time range (96–276 ms) as that of the weight assignment after deviant concatenation. Note that this time range was independently determined in this cohort. The aim of this analysis was to confirm that i) independent setting of weight assignment time performs well for extracting MMR-related components and ii) better SNR is expected in this cohort.

Although the MMR difference sensor waveforms did not show a prominent MMR in either subject (Supplementary Fig. 6A and C) because of low SNR, they still showed MMR with small amplitudes in the bitemporal sensors. $C_{max}$ and $M_{max}$ were evaluated for each component assuming that the reference standard was derived from the same MMR sensors (Supplementary Fig. 6A and C, white circles) and MMR time range (purple lines). Two components showed discriminable $C_{max}$ and $M_{max}$ (Supplementary Fig. 6B and D) and bitemporal topography. These components should be MMR-related components that resemble the reference standard from bitemporal sensors within approximately 100–250 ms, with a similar topographical map and a similar temporal correlation. These results indicated that the generous setting of the weight time range is available as long as the crucial time range (around 100–250 ms) is covered. The finding that only two components were discriminable to maximally resemble the reference standard (i.e., MMR) suggests that MMR-related components were mainly represented by the two components. In terms of SNR, unlike other subjects from the main text (Fig. 6A), it remained challenging to observe higher amplitudes in the decomposed components in this cohort, although they did show a high temporal correlation with the reference standard.

**Supplementary Fig. 1.** The PSD plot of noise-containing repetitive MMR data. The arrow indicates the line peak corresponding to the fundamental frequency. The curly bracket represents the eight harmonics.

**Supplementary Fig. 2.** The results of the spatio-temporal cluster permutation analysis when a different setting of the cluster-defining threshold (*p* = 0.005) was selected. Similar results were obtained using *p* = 0.01.

**Supplementary Fig. 3.** The center of the distribution of the salient components and the slope of the first PCA component superimposed onto the scatter plots of the salient components for individual subjects. The center is indicated by the filled circle. Similar results to those shown in Fig. 7 were revealed at the individual level.

**Supplementary Fig. 4.** Relative contribution (*RC*) results in individual subjects. On the x-axis, *c* represents the index number of the salient component according to the sorting order of the first PCA component axis (Supplementary Fig. 3). Upper panels: *RC* line, lower panels: approximate line according to Eq. (23). Most subjects had a high contribution to the first component in the weighted-*BSS_T/k_* (red lines), and the downslope was steep, arriving at a 5% threshold (gray dotted lines). Count results above the 5% threshold are shown in Table 2. Red, green, and yellow arrows indicate the corresponding components from Subject 2 in Fig. 4.

**Supplementary Fig. 5.** Additional analysis using standard epochs from Subject 7. (**A**) The ERF from sensor space analysis ($\overline{x}_{std}$). Purple line, MMR time (96–276 ms); red line, 300–500 ms. (**B**) The ERF from the weighted-*BSS_T/k_* with a weight on the MMR time (upper) and the scatter plot of $M_{max}$ and $C_{max}$ for each component (lower). No MMR-related components were observed. (**C**) ERF from weighted-*BSS_T/k_* with a weight at 300–500 ms. No outstanding components were observed. The color maps indicate the value of $C_{max}$. Blue and red shading indicate the corresponding weight times.

**Supplementary Fig. 6.** Additional analysis in a new cohort from two subjects. (**A, C**) Sensor space analysis using subtraction for each subject. Red and pink lines from MMR sensors (white circles) within the MMR time (purple line) refer to the reference standard. (**B, D**) The weighted-*BSS_T/k_* results. Upper panels are the source waveforms ($\overline{s}_{sub} or \overline{s}^{\varphi}$), and the lower panels are the scatter plots of $M_{max}$ and $C_{max}$ for each component. The color map in the scatter plot indicates the value of $C_{max}$ (from 0 to 1). Two discriminable components are depicted in red and green with their corresponding topographical maps. In the scatter plots, the arrows with the same color correspond to the components.
